# Photon–photon chemical thermodynamics of frequency conversion processes in highly multimode systems

**DOI:** 10.1038/s41377-025-01856-4

**Published:** 2025-05-12

**Authors:** Huizhong Ren, Georgios G. Pyrialakos, Qi Zhong, Fan O. Wu, Mercedeh Khajavikhan, Demetrios N. Christodoulides

**Affiliations:** 1 Ming Hsieh Department of Electrical and Computer Engineering, University of Southern California, Los Angeles, CA 90089 USA; 2 CREOL, College of Optics and Photonics, University of Central Florida, Orlando, FL 32816 USA; 3 Department of Physics and Astronomy, University of Southern California, Los Angeles, CA 90089 USA; 4 Present Address: School of Applied and Engineering Physics, Cornell University, Ithaca, NY 14853 USA

**Keywords:** Nonlinear optics, Optical physics

## Abstract

Frequency generation in highly multimode nonlinear optical systems is inherently a complex process, giving rise to an exceedingly convoluted landscape of evolution dynamics. While predicting and controlling the global conversion efficiencies in such nonlinear environments has long been considered impossible, here, we formally address this challenge even in scenarios involving a very large number of spatial modes. By utilizing fundamental notions from optical statistical mechanics, we develop a universal theoretical framework that effectively treats all frequency components as chemical reactants/products, capable of undergoing optical thermodynamic reactions facilitated by a variety of multi-wave mixing effects. These photon–photon reactions are governed by conservation laws that directly determine the optical temperatures and chemical potentials of the ensued chemical equilibria for each frequency species. In this context, we develop a comprehensive stoichiometric model and formally derive an expression that relates the chemical potentials to the optical stoichiometric coefficients, in a manner akin to atomic/molecular chemical reactions. This advancement unlocks new predictive capabilities that can facilitate the optimization of frequency generation in highly multimode photonic arrangements, surpassing the limitations of conventional schemes that rely exclusively on nonlinear optical dynamics. Notably, we identify a universal regime of Rayleigh–Jeans thermalization where an optical reaction at near-zero optical temperatures can promote the complete and entropically irreversible conversion of light to the fundamental mode at a target frequency. Our theoretical results are corroborated by numerical simulations in settings where second-harmonic generation, sum-frequency generation and four-wave mixing processes can manifest.

## Introduction

Leveraging nonlinearity for frequency generation^1–13^ has become a central focus in photonic science, enabling groundbreaking advances such as tunable frequency comb sources^14–23^, atomic clocks^24,25^, and sum frequency generation spectroscopies^26,27^, to mention a few. These innovations find numerous applications, in areas ranging from optical communications^28^ and metrology^29,30^ to imaging^31–33^ and bio-photonics^34,35^. To date, frequency generation processes have been predominantly studied in systems with a limited number of transverse modes per frequency component^36–40^. This highlights a vast, unexplored territory in highly multimode structures, such as multimode fibers, nonlinear waveguide arrays and cavities, where the potential for frequency generation remains largely untapped, especially for high-power applications^41,42^. In this respect, exploring novel methodologies to adeptly understand, predict and control the frequency conversion dynamics in such multimode nonlinear settings could lead to new advancements in optics. However, in these environments, optical nonlinearities can promote an exchange of optical power amongst hundreds or thousands of transverse modes and across multiple phase-matched paths, making it almost impossible to engineer the power flow between frequencies and prompting one to wonder if these systems can ever be tamed.

For years, it has been speculated that statistical mechanics could offer a route for deciphering the dynamics of multimode nonlinear optical systems. However, it is only recently that a self-consistent theoretical framework has been put forward^43–45^, offering a new perspective on an array of complex nonlinear phenomena, including beam self-cleaning^46–48^ and beam cooling schemes^43,49^. Grounded in entropic principles, this theory establishes a universal equation of state by means of which the ensued Rayleigh–Jeans (RJ) equilibria can be uniquely determined from the invariant quantities of the system, and consequently for arbitrary excitation conditions^43,45^. On this front, recent experimental studies have provided direct evidence of single-frequency RJ thermalization as well as other archetypical thermodynamic processes in multimode fibers^50^ and time-synthetic photonic mesh lattices^51^. Moreover, further theoretical and experimental advancements have extended this framework to encompass more complex scenarios, including configurations with orbital angular momentum (OAM)^52^ and non-Hermitian settings^53^. This progress now paves the way for the exploration of similar phenomena across multiple degrees of freedom, aiming to provide an altogether new understanding of frequency conversion processes in highly multimode photonic arrangements. A longstanding question in this context is whether heavily multimode settings can achieve complete conversion to a target frequency, akin to single mode optical systems, thus facilitating frequency generation at significantly higher power levels.

In this paper, we show that frequency generation in spatially multimode structures is inherently an all-optical thermodynamic process. In this context, all interacting frequency components can be viewed as photonic “chemical reactants”, capable of undergoing thermodynamic reactions driven by optical nonlinearities. In accord with the second law of thermodynamics, the maximization of optical entropy across all frequencies, leads to RJ equilibria that can be predicted from any possible excitation condition^43–45^. We address this problem in the most general sense by introducing a stoichiometric model that encompasses all possible frequency conversion processes such as difference-frequency generation (DFG), sum-frequency generation (SFG) and four-wave mixing (FWM). Ultimately, we develop a comprehensive strategy to optimize conversion efficiencies in highly multimode environments, offering a promising pathway to enable platforms like parametric oscillators and comb sources at power levels otherwise inaccessible in single-mode configurations^41,42^. In this context, we identify a regime of RJ thermalization, where a reaction at near-zero temperatures not only maximizes frequency conversion but also promotes the entropically irreversible funneling of power to the fundamental mode at the target frequency. These optimization capabilities are underpinned by a key result in our study: an expression that formally relates the stoichiometric coefficients to the optical chemical potentials, in full analogy to what is expected in molecular/atomic chemical reactions, where the Gibbs free energy is extremized^54,55^. Our theoretical framework can offer unique insight into the long-term dynamics of systems supporting hundreds or thousands of spatiotemporal modes, paving the way for novel methodologies aimed at controlling and harnessing their frequency conversion capabilities.

## Results

### Theory

To formulate the aspects governing these optical “chemical reactions”, we begin by considering an exemplary frequency generation process in an arbitrary multimoded optical system. In the case under consideration, let us assume that the reaction involves four frequencies, the exchange of which is written in a “stoichiometric” fashion according to

1 $${\nu }_{1}{\omega }_{1}+{\nu }_{2}{\omega }_{2}\rightleftharpoons {\nu }_{3}{\omega }_{3}+{\nu }_{4}{\omega }_{4}$$

where ${\omega }_{1}$ and ${\omega }_{2}$ effectively correspond to optical reactants, while ${\omega }_{3}$ and ${\omega }_{4}$ represent the product species. The integers ${\nu }_{k}$ associated with each corresponding frequency ${\omega }_{k}$ represent stoichiometric coefficients in this optical reaction, determined by the respective laws of energy conservation. Equation (1) can capture a variety of frequency conversion phenomena ranging from second-harmonic generation (SHG) to FWM effects. Under such conditions, the nonlinear evolution of the optical field |*Ψ*_*k*_〉, associated with a frequency species *ω*_*k*_, is governed by

2 $$\frac{{id}{|\varPsi }_{k}{{\rangle }}}{{dz}}=-\left({\widehat{H}}_{L,k}+{\widehat{H}}_{{NL},k}\right){|\varPsi }_{k}{{\rangle }}$$

where ${\widehat{H}}_{L,k}$ denotes a linear propagation operator. In discrete optical systems, like multicore fibers and arrays, ${\widehat{H}}_{L,k}$ has the form of a coupling matrix, while in multimode optical waveguides such as fibers, ${\widehat{H}}_{L,k}={\partial }_{x}^{2}+{\partial }_{y}^{2}+{V}_{k}\left(x,y\right)$, where the scaled function ${V}_{k}\left(x,y\right)$ is proportional to the waveguide’s index profile. In all cases, the eigenspectrum and eigenfunctions of the ${\widehat{H}}_{L,k}$ operator correspond to the propagation constants ${\epsilon }_{k,i}$ and the guided modes $|{\psi }_{k,i}\rangle$ supported in the waveguide structure, respectively. Finally, the operator ${\widehat{H}}_{{NL},k}$ arises from the underlying nonlinear process that is responsible for the multi-wave mixing effects as will be discussed shortly.

To describe frequency conversion phenomena through the lens of optical thermodynamics, it is essential to identify the first three pertinent conservation laws dictating the multi-frequency balance in the optical reaction described in the specific example of Eq. (1). These conservation laws or Manley–Rowe relations^36^ always result from a hidden symmetry in the system (see Supplementary S.A). For the case considered in Eq. (1), the first invariant (Manley–Rowe relation), ${N}_{1}={P}_{1}/{\nu }_{1}-{P}_{2}/{\nu }_{2}$, indicates that, under the stoichiometric numbers of Eq. (1), the destruction of ${\nu }_{1}$ wave packets (or photons) at ${\omega }_{1}$ will be accompanied by an annihilation of ${\nu }_{2}$ photons at ${\omega }_{2}$. For the remaining frequency components, the relations ${N}_{2}={P}_{1}/{\nu }_{1}+{P}_{3}/{\nu }_{3}$ and ${N}_{3}={P}_{1}/{\nu }_{1}+{P}_{4}/{\nu }_{4}$ similarly introduce two additional constants of motion. In the above expressions, the normalized power levels ${P}_{k}=\mathop{\sum}\limits_{i=1}^{{M}_{k}}{|{c}_{k,i}\left(z\right)|}^{2}$ represent the power carried by a frequency component $k$, supporting a total of ${M}_{k}$ guided transverse modes. Here, ${c}_{k,i}\left(z\right)$ stands for the complex modal amplitude of mode $i$, as obtained via a projection of the optical field state vector ${|\varPsi }_{k}\left(z\right)\rangle$ on the transverse mode field profile $|{\psi }_{k,i}\rangle$, at a propagation distance $z$. The $z$-dependance of the modal occupancies ${\left|{c}_{k,i}\left(z\right)\right|}^{2}={|\langle {\psi }_{k,i}{|\varPsi }_{k}\left(z\right)\rangle |}^{2}$ is a direct consequence of nonlinearity, that promotes the chaotic exchange of power among all transverse modes and frequencies. Finally, wave propagation in nonlinear multimode waveguides exhibits an additional invariant. This fourth conserved quantity is associated with the “kinetic” or internal energy $U=-\mathop{\sum}\nolimits_{k}\langle {\varPsi }_{k}|{\hat{H}}_{L,k}|{\varPsi }_{k}\rangle =-\mathop{\sum}\nolimits_{k}\mathop{\sum}\nolimits_{i}{\epsilon }_{k,i}{|{c}_{k,i}|}^{2}$, where ${\epsilon }_{k,i}$ denotes the eigenvalue (propagation constant) of the *i*th transverse mode at frequency ${\omega }_{k}$. One can formally show that this last invariant *U* is equivalent to the conservation of the Minkowski electrodynamic momentum ${T}_{{zz}}$ ^56^ over all frequency components. We emphasize that both the material’s optical properties and the structure’s geometric characteristics are implicitly reflected in the eigenvalue spectrum ${\epsilon }_{k,i}$.

To frame this complex process as a thermodynamic photon–photon chemical reaction, we utilize the fact that in a multimode setting, the nonlinear dynamics described by Eq. (2) unfold in a chaotic and thus ergodic fashion. In our formalism, we assume a relatively weak nonlinearity, under which frequency components behave like “gaseous” entities, thus prohibiting the formation of coherent objects such as solitons. In this context the role of nonlinearity is dual: it facilitates a continuous and chaotic exchange of optical power among all ${\sum }_{k=1}^{4}{M}_{k}$ guided modes, across the four interacting frequency species while ensuring that, over time, the system will explore in a fair manner its entire phase space, constrained by the four invariants (${N}_{1},{N}_{2},{N}_{3},U$). Notably, these four quantities are directly determined by the modal occupancies ${J}_{k,i}\equiv {\left|{c}_{k,i}\right|}^{2}$, an aspect that is crucial for rigorously deriving the equilibrium states of light in such convoluted multimode configurations. To do so, we use the Boltzmann entropy

3 $$S=\mathop{\sum}\limits_{k}\mathop{\sum}\limits_{i=1}^{{M}_{k}}{\mathrm{ln}}{J}_{k,i}$$

that is appropriate for this classical micro-canonical system^43,44,57–65^. At equilibrium, the average power levels $\left\langle {P}_{k}\right\rangle$ associated with the optical reactants and products can then be obtained by maximizing the total entropy of the system $S$, as expected from the second law of thermodynamics. In the presence of the four invariants, this can be achieved by deploying four Lagrange multipliers. This procedure, as outlined in the “Materials and methods” section, reveals that at frequency ${\omega }_{k}$, upon thermalization, the average power occupancy of a transverse mode $i$ will obey a RJ distribution:

4 $$\left\langle {J}_{k,i}\right\rangle =-\frac{T}{{\epsilon }_{k,i}+{\mu }_{k}}$$

where $T$ represents a common optical temperature and ${\mu }_{k}$ the corresponding optical chemical potential that happens to be different for each frequency component. Importantly, in this case, one can rigorously prove that the chemical potentials pertaining to each frequency, are related through (see “Materials and methods”)

5 $${\nu }_{1}{\mu }_{1}+{\nu }_{2}{\mu }_{2}={\nu }_{3}{\mu }_{3}+{\nu }_{4}{\mu }_{4}$$

In this context, the equilibrium conditions for this utterly complex multimode, multi-frequency conversion process can be established: each frequency component attains its own RJ distribution, characterized by a global optical temperature but distinct chemical potentials (Eq. (4)). Meanwhile, the chemical potentials are balanced through the stoichiometric coefficients ${\nu }_{1-4}$ as indicated by Eq. (5). Notably, Eq. (5) is formally analogous to what one may expect in actual multi-species molecular/atomic chemical reactions, where extremization of the Gibbs free energy leads again to ${\sum }_{j}{\nu }_{j}{\mu }_{j}=0$^54,55^, formally relating the stoichiometric coefficients to the chemical potentials. Clearly, the role of stoichiometry in the chemical relations (Eq. (5)) should have been anticipated given that both settings (chemical and optical) are governed by the laws of statistical mechanics. Importantly, using the RJ distribution, the internal energy ${U}_{k}$ and the optical power ${P}_{k}$ associated with each frequency component ${\omega }_{k}$, one can formally derive the following equation of state (see “Materials and methods”)

6 $${U}_{k}-{\mu }_{k}{P}_{k}={M}_{k}T$$

which relates the intensive variables $T$ and ${\mu }_{k}$ to three extensive quantities ${U}_{k}$, ${M}_{k}$, and ${P}_{k}$.

Equations (5) and (6) now provide a versatile and powerful tool for predicting the equilibrium power distribution of each frequency component in such complex multimode systems. Specifically, they enable one to uniquely determine the key thermodynamic quantities $T,{\mu }_{1-4}$ and consequently the RJ distributions at equilibrium (Eq. (4)), directly from the excitation conditions of the guiding system (see Supplementary S.E). As we will demonstrate in subsequent sections, this predictive capability can be leveraged to optimize the design parameters of a highly multimoded waveguide arrangement, thus achieving near 100% conversion efficiency at a target frequency. Clearly, to realistically achieve this goal, the nonlinear material should display high transparency over the spectral range spanning the frequency conversion processes.

**Fig. 1:**
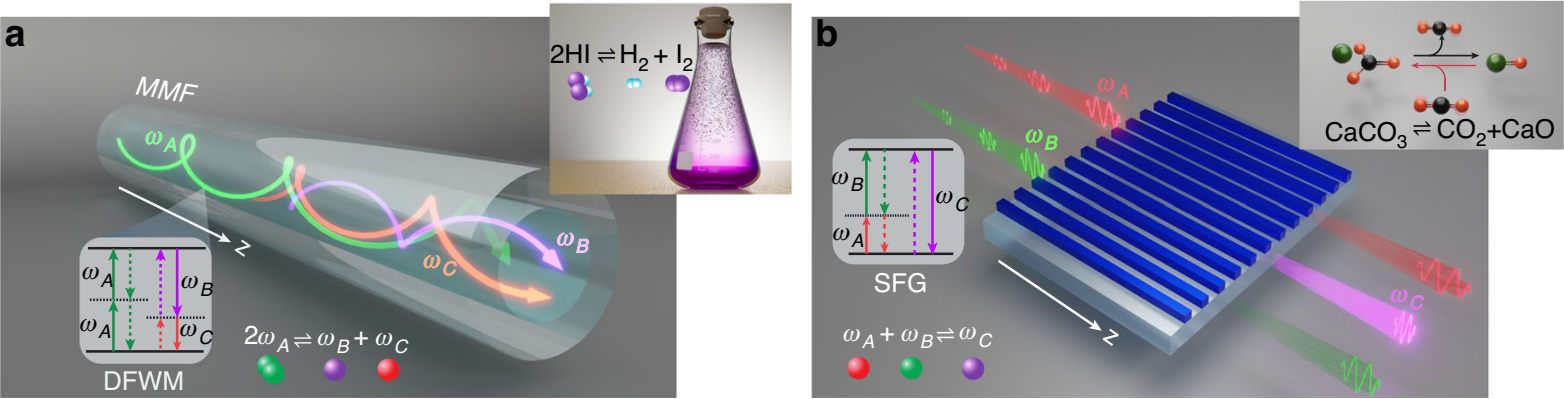
**Frequency conversion thermodynamic processes in continuous and discrete photonic arrangements. a** Schematic of a degenerate four wave-mixing process $2{\omega }_{A}\rightleftharpoons {\omega }_{B}+{\omega }_{C}$, in a ${\chi }^{\left(3\right)}$ graded-index (GRIN) multimode silica fiber. Here, the stoichiometry is mathematically analogous to the reversible reaction of hydrogen iodide $2{\rm{HI}}\rightleftharpoons {{\rm{H}}}_{2}+{{\rm{I}}}_{2}$. **b** Sum-frequency generation process ${\omega }_{A}+{\omega }_{B}\rightleftharpoons {\omega }_{C}$ in a ${\chi }^{\left(2\right)}$ LiNbO_3_ nonlinear waveguide lattice, which is conceptually resembling for example thermal decomposition of calcium carbonate, i.e., ${{\rm{CaCO}}}_{3}\rightleftharpoons {\rm{CaO}}+{\rm{C}}{{\rm{O}}}_{2}$

**Fig. 2:**
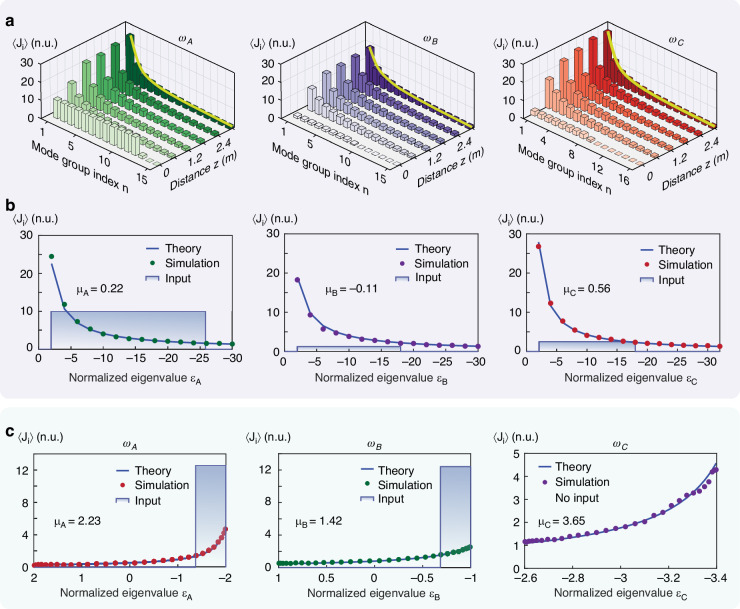
**Thermalization dynamics of frequency conversion processes in continuous and discrete photonic arrangements. a** Evolution of the average transverse modal occupancies $\langle {J}_{i}\rangle$ for the three frequency components interacting via degenerate four-wave mixing in the GRIN fiber (Fig. 1a). Here, the fiber core radius is 31.25 µm, with a refractive index contrast of $\left({n}_{{core}}-{n}_{{clad}}\right)/{n}_{{core}}=0.01$. The core refractive index ${n}_{{core}}$ is calculated using the Sellmeier equation for silica. The system is initiated by exciting equally a subset of transverse modes, primarily at frequency ${\omega }_{A}$. **b** Comparison between simulation results and theoretical predictions. The resulting RJ equilibria exhibit a common temperature $T=0.25$ and distinct chemical potentials, ${\mu }_{A}=0.22$, ${\mu }_{B}=-0.11$, and ${\mu }_{C}=0.56$, in accord with Eq. (5). **c** Average modal distributions at thermal equilibrium for a LiNbO_3_ lattice configuration involving 30 waveguide elements as depicted in Fig. 1b. The corresponding theoretical temperature and chemical potentials are predicted to be $T=-0.01$ and ${\mu }_{A}=2.23$, ${\mu }_{B}=1.42$, and ${\mu }_{C}=3.65$, resulting in RJ equilibria that match perfectly with numerical simulation results. Here, a negative temperature indicates that higher-order modes are favored

To corroborate our theoretical analysis, we investigate the optical thermodynamics of two distinct frequency generation processes. First, we examine a degenerate four-wave mixing process unfolding in a weakly guiding, ${\chi }^{\left(3\right)}$ nonlinear silica multimode fiber, as illustrated in Fig. 1a. This frequency conversion process, described by $2{\omega }_{A}\rightleftharpoons {\omega }_{B}+{\omega }_{C}$, is for example conceptually analogous to the reversible decomposition of hydrogen iodide: $2{\rm{HI}}\rightleftharpoons {{\rm{H}}}_{2}+{{\rm{I}}}_{2}$, characterized by stoichiometric coefficients ${\nu }_{1}=2$, ${\nu }_{2}=0$, ${\nu }_{3}={\nu }_{4}=1$. According to Eq. (5), the chemical potentials associated with each frequency component must satisfy $2{\mu }_{A}={\mu }_{B}+{\mu }_{C}$. In our example, the wave-mixing process involves three wavelengths, ${\lambda }_{A}=1272\,{\rm{nm}}$, ${\lambda }_{B}=1306\,{\rm{nm}}$, and ${\lambda }_{C}=1240\,{\rm{nm}}$, with the fiber supporting a total number of ${M}_{A}=120,{M}_{B}=120$ and ${M}_{C}=136$ guided transverse modes at each respective wavelength^66^. The individual propagation constants of all guided modes are rigorously calculated from the fiber parameters listed in the caption of Fig. 2, taking also into account the wavelength-dependence of silica glass. Let us consider for example, a situation where the fiber is excited at all three wavelengths with powers ${P}_{A}=800\,{\rm{kW}},\,{P}_{B}=50\,{\rm{kW}},\,{P}_{C}=100\,{\rm{kW}}$. By carefully shaping the transverse profiles of the injected beams, the normalized total internal energy in this system is $U=1.532\times {10}^{4}$, dictated by the initial power distribution among modes. These input conditions provide direct knowledge of the four invariants quantities (${N}_{1},{N}_{2},{N}_{3},U$), from where one can analytically predict the intensive parameters of the chemical equilibrium state of Eq. (4), that happen to be $T=0.25$, ${\mu }_{A}=0.22$, ${\mu }_{B}=-0.11$, ${\mu }_{C}=0.56$ (see Supplementary S.E). To monitor the thermalization dynamics of light in this multimode fiber, we numerically simulate the nonlinear dynamics of all the modes involved under continuous wave (CW) or broad pulse conditions (see Supplementary S.B). These results are depicted in Fig. 2a, b, clearly demonstrating that the averaged power modal occupancies eventually settle to RJ distributions after $3\,{\rm{m}}$ of propagation, in excellent agreement with our theoretical predictions, i.e., Eq. (4).

To further demonstrate the predictive capabilities of our theory, we study a different frequency generation process by carrying out simulations on a multicore ${\chi }^{\left(2\right)}$ nonlinear optical waveguide array^13,67–69^. Specifically, we investigate a LiNbO_3_ lattice involving $M=30$ identical guide elements as schematically depicted in Fig. 1b. The linear eigenvalues ${\epsilon }_{k,i}$ corresponding to the *i*th optical supermode of the lattice at frequency ${\omega }_{k}$ are given by ${\epsilon }_{k,i}=2{\kappa }_{k}\cos [\pi i/(M+1)]+{\beta }_{k}$, where ${\kappa }_{k}$ stands for the uniform coupling coefficient between two neighboring waveguide elements, and ${\beta }_{k}$ denotes the propagation constant of the local mode at each individual site. Note that all the parameters are defined at the frequency ${\omega }_{k}$. In this type-0 ${\chi }^{\left(2\right)}$ configuration, sum-frequency generation can occur, described by the stoichiometric relation ${\omega }_{A}+{\omega }_{B}\rightleftharpoons {\omega }_{C}$, which is conceptually analogous to that describing say the thermal decomposition of calcium carbonate, i.e., ${{\rm{CaCO}}}_{3}\rightleftharpoons {\rm{CaO}}+{{\rm{CO}}}_{2}$. Consequently, at thermal equilibrium, the chemical potentials are expected to satisfy ${\mu }_{A}+{\mu }_{B}={\mu }_{C}$. In simulating the discrete coupled evolution equations, the nearest-neighbor normalized coupling coefficients for these three frequencies are here taken as ${\kappa }_{A}=1$, ${\kappa }_{B}=0.5$, ${\kappa }_{C}=0.2$, with a scaled phase mismatch of $\Delta ={\beta }_{C}-{\beta }_{A}-{\beta }_{B}=-3$ (see Supplementary S.D). Accordingly, the normalized propagation constants for the three components span the ranges $-2\le {\epsilon }_{A}\le 2$, $-1\le {\epsilon }_{B}\le 1$ and $-3.4\le {\epsilon }_{C}\le -2.6$, respectively. For initial conditions specified in the Fig. 2c, one can predict a global negative temperature $T=-0.01$ with chemical potentials ${\mu }_{A}=2.23$, ${\mu }_{B}=1.42$, and ${\mu }_{C}=3.65$ (see Supplementary S.E). Numerical simulations, depicted in Fig. 2c, corroborate our theoretical predictions.

### Optimizing frequency conversion efficiency in multimode systems

The theory presented herein provides a fundamentally new perspective on the dynamic behavior of multimode multifrequency photonic systems, revealing a large untapped potential for not only optimizing but also harnessing their frequency conversion capabilities. Notably, the predictive strength of our all-optical chemical thermodynamic framework enables the systematic design of highly multimoded photonic arrays which as we will see, in principle, can achieve nearly 100% conversion efficiencies at a target frequency—a prospect previously restricted to single- or few-mode settings. Our approach to optimizing frequency conversion is grounded in the principle that the equilibrium state of a “chemical” conversion process is explicitly determined by the relative spectral properties of the reactants and products. In such thermalizing multi-frequency systems, the four invariant quantities ${N}_{1}$, ${N}_{2}$, ${N}_{3}$, and $U$—which can be derived from initial excitation conditions and the propagation constants of the guiding system—are always associated with a unique solution for ($T,{\mu }_{1},{\mu }_{2},{\mu }_{3}$). This aspect provides immediate knowledge of the equilibrium states across any physical or excitation parameter, thus revealing parametric regimes where long-term conversion efficiencies are maximized. In this regard, the conversion efficiency into a designated frequency product can be exceptionally high when initial excitation conditions position the system (to the extent the experiment allows) to attain a near-zero optical temperature once thermal equilibrium is reached. Under these conditions, not only is the conversion highly efficient, but the energy also settles into the fundamental mode of the target frequency.

**Fig. 3:**
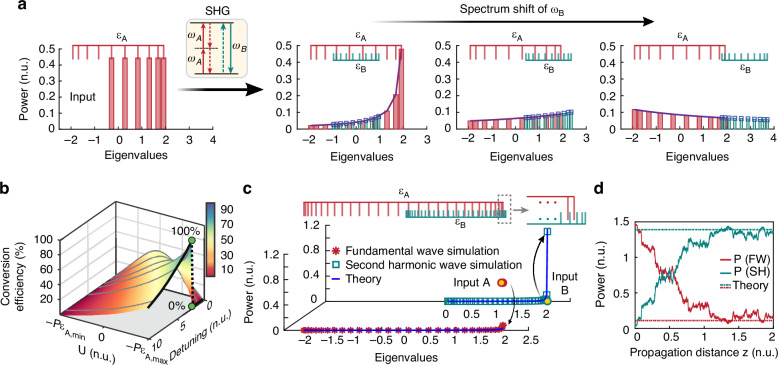
**Optimizing frequency conversion for second-harmonic generation. a** Thermal equilibrium states of a SHG optical reaction occurring in a LiNbO_3_ lattice involving ten waveguide elements. For all three cases, the system is excited with the same power, shared between the six lower order modes (left panel). As the phase mismatch between the fundamental and harmonic wave increases, the system transits from a positive temperature to a negative temperature regime, where the higher order modes are preferentially populated. The equilibrium power ratios between the SH and FW (${P}_{B}/{P}_{A}$) are 0.44, 1.25, 0.7 for the three cases respectively. **b** Theoretically obtained conversion efficiency as a function of input energy $U$ and phase mismatch $\Delta$ for a LiNbO_3_ lattice involving 30 elements. **c** Numerically simulated equilibrium power distributions at the maximum conversion efficiency point, marked by the green dot in **b**. The first two modes of the FW are evenly excited with a total power of ${P}_{A}=1.5$ while the two frequencies exhibit a phase mismatch of $\Delta =2.1$. At thermal equilibrium, the system reaches a near-zero temperature RJ state, with a theoretically predicted $T=0.0018$, matching simulations results. **d** Optical power evolution as obtained from numerical simulations for the latter case. After thermalization, a conversion efficiency of 91% is attained, in agreement with theoretical predictions

To demonstrate this aspect in a realistic optical setting^13,67–69^, we first consider an example involving SHG in a LiNbO_3_ lattice comprising *M* = 10 sites, as shown in Fig. 1b. The stoichiometric relation characterizing this process is $2{\omega }_{A}\rightleftharpoons {\omega }_{B}$, where ${\omega }_{A}$ and ${\omega }_{B}$ represent the frequencies of the fundamental wave (FW) and second-harmonic wave (SH), respectively. The linear eigenvalues (normalized propagation constants) for these two frequency components are ${\epsilon }_{A,i}=2{\kappa }_{A}\cos [\pi i/(M+1)]$ and ${\epsilon }_{B,i}=2{\kappa }_{B}\cos [\pi i/(M+1)]+\Delta$, where $\Delta =2{\beta }_{A}-{\beta }_{B}$ is a phase mismatch and ${\kappa }_{A}={\kappa }_{B}=1$. In this example, the reaction takes place under only two conservation laws, namely ${N}_{1}={\sum }_{i=1}^{M}\left({J}_{A,i}/2+{J}_{B,i}\right)$ and $U=-{\sum }_{i=1}^{M}\left({\epsilon }_{A,i}{J}_{A,i}+{\epsilon }_{B,i}{J}_{B,i}\right)$. Here, for visualization purposes, we renormalize the spectrum, ${\epsilon }_{B,i}^{{\prime} }={\epsilon }_{B,i}/2$, and the power ${J}_{B,i}^{{\prime} }=2{J}_{B,i}$ of species $B$, leading to ${N}_{1}={\sum }_{i=1}^{M}\left({J}_{A,i}+{J}_{B,i}^{{\prime} }\right)/2$ and $U=-{\sum }_{i=1}^{M}\left({\epsilon }_{A,i}{J}_{A,i}+{\epsilon }_{B,i}^{{\prime} }{J}_{B,i}^{{\prime} }\right)$. Under these conditions, our theory predicts that, at thermal equilibrium, the average modal occupancies ${J}_{A,i}$ and ${J}_{B,i}^{{\prime} }$ converge to a common RJ distribution with the same optical temperature $T$ and renormalized chemical potentials ${\widetilde{\mu }}_{A}={\widetilde{\mu }}_{B}$. Note that this latter expression exactly corresponds to that expected from Eq. (5), i.e., $2{\mu }_{A}={\mu }_{B}$ before renormalization, thus adhering to the stoichiometry of the model. Figure 3a illustrates a scenario where the LiNbO_3_ lattice is excited at the fundamental frequency by populating the first six transverse modes with equal power. Here, we monitor the equilibria of the system for different values of the phase mismatch parameter $\Delta$ between the FW and SH, which can be typically adjusted by altering the actual sample temperature^13,67–69^. Our analytical predictions reveal that the equilibria can continuously transition from a positive to a negative temperature regime as the renormalized spectrum of the SH ${\epsilon }_{B}^{{\prime} }$ shifts (Fig. 3a). More importantly, we observe that the equilibrium power balance between the FW and SH is directly dictated by the spectral shift leading to an important pertinent question: can we identify an optimal set of initial conditions and design parameters that maximizes the generated SH component?

Figure 3b illustrates the conversion efficiency of the SHG process as a function of the input energy $U$—determined by the initial distribution of power amongst the transverse modes of the FW—and the phase mismatch $\Delta$. In this design, ${\kappa }_{A}={\kappa }_{B}=1$, and the array supports $M=30$ supermodes in both the fundamental and the second harmonic resulting from the first and third local transverse mode at each guiding site, respectively^67^. The conversion efficiency $\left\langle {P}_{B}\right\rangle /{N}_{1}={\sum }_{i=1}^{M}\left\langle {J}_{B,i}\right\rangle /{N}_{1}$ is analytically calculated via Eq. (4) after extracting the thermodynamic quantities $(T,{\mu }_{A},{\mu }_{B})$ from the known parameters $(U,P,{\epsilon }_{A,i},{\epsilon }_{B,i})$ and Eqs. (5) and (6) (see Supplementary S.E). Evidently an abrupt transition from 0% to 100% conversion efficiency, marked by the green dot in Fig. 3b, can take place at a phase mismatch of approximately $\Delta =2.1$. On the $U$ axis, this effect is observed at near-absolute-zero optical temperatures, associated with a minimum energy value of $U=-P{\epsilon }_{A,\max }$, where the FW is almost exclusively excited in its fundamental mode. Typically, at near-zero temperatures, equilibrium conditions promote strong condensation of light into a global ground state shared between the FW and the SH. This transition results from an abrupt shift of the global ground state from the fundamental mode of the FW, associated with a renormalized propagation constants ${\epsilon }_{A,\max }$, to the fundamental mode of the SH, associated with ${\epsilon^{\prime} }_{B,\max }$. It is important to note that, in all cases, light is injected solely in the FW, thus leading to a complete transfer of power to the SH when the detuning is approaching the transition point, following the onset of thermalization.

What is remarkable here is that frequency conversion can still reach maximum efficiency, even in highly complex multimode systems where it’s impossible to perfectly match the phases of all transverse modes in the FW and SH. In such systems, instead, the effect is driven purely by optical thermodynamics, governed by the underlying laws of the respective optical reaction. To numerically validate the theoretically anticipated results of Fig. 3b, we simulate the SHG process at $\Delta =2.1$ (Eqs. (C.2) in the Supplementary S.C) by exciting equally the first two modes of the FW with total normalized power of $P=1.5$ (the corresponding actual power in LiNbO_3_ is $12\,{\rm{W}}$). Figure 3c shows the modal occupancies for both the FW and the SH, at the input and after thermal equilibrium is attained at the output. As Fig. 3c shows, the two species successfully relax to the theoretically predicted RJ equilibria, with $T=0.0018$ and ${\mu }_{A}=-2.046$, while most of the power, initially occupying the FW, is now transferred to the fundamental mode of the SH, resulting in a conversion efficiency of 91%. Figure 3d illustrates the power evolution as a function of the propagation distance, indicating that in this multimode arrangement, power transfer is entropically irreversible.

**Fig. 4:**
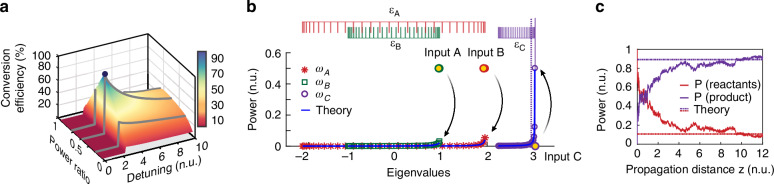
**Optimizing frequency conversion for sum-frequency generation. a** Conversion efficiency of the SFG process as a function of input power ratio and phase mismatch $\varDelta$, for a LiNbO_3_ waveguide array involving 30 waveguide elements. The input energy is set at the optimal zero-temperature value of $U=-{P}_{A}{\epsilon }_{A,\max }-{P}_{B}{\epsilon }_{B,\max }$. **b** Average modal occupancies at equilibrium, obtained via numerical simulation of Eq. (2) at the maximum conversion efficiency point, indicated with a black dot in (**a**). The two reactants ${\omega }_{A}$ and ${\omega }_{B}$ are initially excited with equal powers (${P}_{A}={P}_{B}=1$), each equally distributed in their respective two lower-order modes. In this case, the system thermalizes to a near-zero temperature state with $T=0.0013$, transferring power to the third frequency ${\omega }_{C}$. **c** Evolution of the optical power for the optical reactants (${\omega }_{A}$ or ${\omega }_{B}$) and products (${\omega }_{C}$) as obtained from numerical simulations. After thermalization, a conversion efficiency of 92.3% is achieved, consistent with theoretical predictions

The general methodology outlined here is universal and can be extended to other frequency conversion processes. We next consider a type-0 sum-frequency generation process, ${\omega }_{A}+{\omega }_{B}\rightleftharpoons {\omega }_{C}$, unfolding in a LiNbO_3_ waveguide array, as schematically shown in Fig. 1b. As before, the array supports $M=30$ supermodes in each frequency, resulting from the first (at ${\omega }_{A}$ and ${\omega }_{B}$), and third (${\omega }_{C}$) local transverse mode at each guiding site, respectively. In this configuration, the coupling coefficients associated with frequencies ${\omega }_{A}$, ${\omega }_{B}$ and ${\omega }_{C}$ are taken to be ${\kappa }_{A}=1$, ${\kappa }_{B}=0.5$ and ${\kappa }_{C}=0.2$, respectively (see Supplementary S.G). Consequently, the corresponding eigenvalue spectra at zero detuning will lie in the ranges of $-2\le {\epsilon }_{A}\le 2$, $-1\le {\epsilon }_{B}\le 1$ and $-0.4\le {\epsilon }_{C}\le 0.4$. To study this case, we fix the input internal energy at $U=-{P}_{A}{\epsilon }_{A,\max }-{P}_{B}{\epsilon }_{B,\max }$, associated with the optimal zero-temperature equilibrium state for the SFG process. Figure 4a displays the conversion efficiency at the generated frequency ${\omega }_{C}$ as a function of the power ratio ${P}_{B}/{P}_{A}$ of the reactants and the phase mismatch parameter $\Delta ={\beta }_{C}-{\beta }_{B}-{\beta }_{A}$. As predicted by our chemical thermodynamic formalism and illustrated in Fig. 4a, the conversion efficiency attains a maximum at an input power ratio of ${P}_{A}:{P}_{B}=1:1$ and a critical phase mismatch of approximately $\Delta =-({\epsilon }_{C,\max }-{\epsilon }_{A,\max }-{\epsilon }_{B,\max })=2.6$. To validate this theoretical result, we performed numerical simulations over a range of parameters from where we found that indeed at a phase mismatch of $\Delta \approx 2.68$, and input powers ${P}_{A}={P}_{B}=1$ (actual powers for ${\lambda }_{A}$ and ${\lambda }_{B}$ are 2.9 W and 3.4 W, respectively), the conversion efficiency is maximized when the input power is equally shared between the first two modes of each frequency component (${\omega }_{A}$, ${\omega }_{B}$). As shown in Fig. 4b, at thermal equilibrium, the optical power is irreversibly and almost entirely transferred from the reactant frequencies ${\omega }_{A}$ and ${\omega }_{B}$ to the product frequency ${\omega }_{C}$, occupying mostly the fundamental mode at ${\omega }_{C}$. This state is characterized by three near-zero temperature RJ distributions with parameters $T=0.0013$, ${\mu }_{A}=-2.022$, ${\mu }_{B}=-1.058$ and ${\mu }_{C}=-3.08$, as accurately predicted by our theory. Figure 4c depicts the power evolution along the propagation axis (as obtained numerically by solving Eqs. (D.2) in the Supplementary S.D), demonstrating an entropically irreversible conversion of power to ${\omega }_{C}$ with an efficiency of 92.3%.

**Fig. 5:**
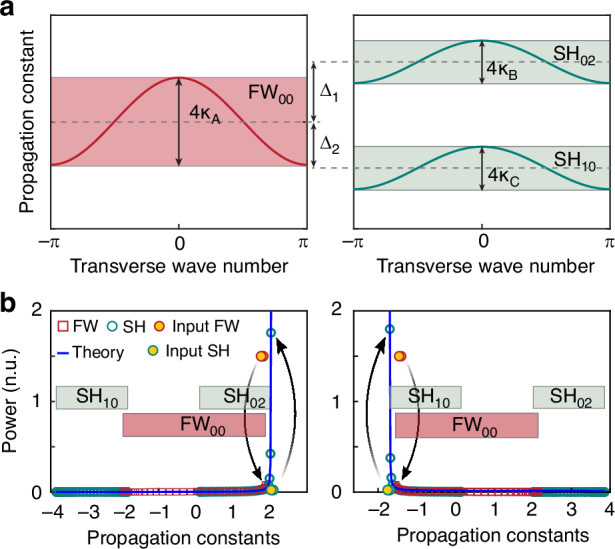
**Second-harmonic generation when two mode bands are involved at the second harmonic. a** Eigenvalue bands corresponding to the FW (left panel) and the SH (right panel) waves. Note that ${\kappa }_{A,B,C}$ denote the coupling coefficients associated with the local modes for the FW and SH waves, while ${\varDelta }_{1},{\varDelta }_{2}$ represent the phase mismatch between the FW and SH_02_, SH_10_ eigenvalue band. **b** By judiciously varying either the initial FW excitation conditions or the detuning parameters, the majority of the FW optical power can be funneled towards either the SH_02_ band (left panel) or the SH_10_ band (right panel). In all instances, the numerical simulations show excellent agreement with the theoretical predictions

Of interest would be exploring more convoluted waveguide array configurations where the local FW fundamental spatial mode is nonlinearly coupled to two higher-order local even modes at the second harmonic frequency that only nonlinearly interact via the FW^68,70,71^ as shown in Fig. 5a. This system can be implemented in a periodically poled lithium niobate waveguide lattice as detailed in^68^. Note that the poling features a single period, which does not enable phase-matching for all the modes involved. In this scenario, as Fig. 5b indicates, at thermal equilibrium, the two frequency components reach their own RJ distribution at the same optical temperature, while the chemical potentials are again balanced, with $2{\mu }_{A}={\mu }_{B}$. The same optimization strategy can also be employed to transfer optical power from the fundamental wave to either the lowest or highest supermode within a specific band at the second harmonic.

## Conclusions and discussion

In conclusion, we have developed a comprehensive theoretical framework that accurately predicts the statistics of frequency conversion processes in nonlinear highly multimode optical environments. By leveraging principles from statistical mechanics, we introduced a stoichiometric model that, along with the accompanying conservation laws, provides a comprehensive method for calculating the average occupancies of the transverse modes at each frequency once RJ equilibria are established. Our theory indicates that, upon thermalization, all frequency components attain the same optical temperature and distinct chemical potentials, uniquely determined by the initial conditions. Notably, the equilibrium balance of chemical potentials is dictated by a universal law that directly reflects the stoichiometry of the optical frequency conversion, in a way akin to molecular and atomic chemical reactions. This result enables us to identify a parametric regime where an optical multi-frequency reaction at near-zero optical temperature irreversibly maximizes frequency conversion across the entire spatial spectrum. The work presented here may open new possibilities in controlling and harnessing complex interactions in high-power multimode optical parametric oscillators and amplifiers as well as in bio-imaging applications.

Finally, we would like to emphasize that it remains uncertain to what extent the correspondence between chemical thermodynamics and optical thermodynamics holds. The justification for this premise rests on two key points: (i) the fact that the different optical species (frequencies) eventually settle at thermal equilibrium into the same temperature in their corresponding Rayleigh-Jeans distributions and, (ii) the chemical potentials are stoichiometrically related as in chemistry, given that the exchange of photons should satisfy the pertinent Manley–Rowe relations. In addition, in the microcanonical setting discussed here, the frequency conversion processes happen to be strictly speaking amphidromous unless of course the system is placed (under appropriate initial conditions) at a near zero optical temperature in which case the conversion into a predesignated frequency is thermodynamically irreversible. At this stage, one might also naturally ask whether these all-optical processes could also be described using other thermodynamic quantities, such as enthalpy or Gibbs free energy, or whether overarching principles like that of Le–Chatelier still apply.

## Materials and methods

### Chemical potential relation

Here we analytically derive the chemical potential balance relation, as expressed by Eq. (5) in the main text. Once optical thermal equilibrium is attained, the entropy associated with a frequency conversion process will reach a maximum, subject to constraints imposed by the conservation laws:

$${N}_{1}=\frac{1}{{\nu }_{1}}\mathop{\sum }\limits_{i=1}^{{M}_{1}}{J}_{1,i}-\frac{1}{{\nu }_{2}}\mathop{\sum }\limits_{i=1}^{{M}_{2}}{J}_{2,i}$$

$${N}_{2}=\frac{1}{{\nu }_{1}}\mathop{\sum }\limits_{i=1}^{{M}_{1}}{J}_{1,i}+\frac{1}{{\nu }_{3}}\mathop{\sum }\limits_{i=1}^{{M}_{3}}{J}_{3,i}$$

$${N}_{3}=\frac{1}{{\nu }_{1}}\mathop{\sum }\limits_{i=1}^{{M}_{1}}{J}_{1,i}+\frac{1}{{\nu }_{4}}\mathop{\sum }\limits_{i=1}^{{M}_{4}}{J}_{4,i}$$

$$U=-\mathop{\sum }\limits_{k=1}^{4}\mathop{\sum }\limits_{i=1}^{{M}_{k}}{\epsilon }_{k,i}{J}_{k,i}$$

where ${J}_{k,i}\equiv \langle {\left|{c}_{k,i}\right|}^{2}\rangle$ denotes the average modal occupancy of the *i*th spatial mode at frequency ${\omega }_{k}$. Extremization by means of four Lagrange multipliers $\alpha ,\beta ,\gamma$ and $\delta$ leads to:

$$\begin{array}{l}\frac{\partial }{\partial {J}_{k,i}}\left\{S+\alpha \left[\left(\frac{1}{{\nu }_{1}}\mathop{\sum }\limits_{i=1}^{{M}_{1}}{J}_{1,i}-\frac{1}{{\nu }_{2}}\mathop{\sum }\limits_{i=1}^{{M}_{2}}{J}_{2,i}\right)-{N}_{1}\right]+\beta \left[\left(\mathop{\sum }\limits_{k=1}^{4}\mathop{\sum }\limits_{i=1}^{{M}_{k}}{\epsilon }_{k,i}{J}_{k,i}\right)+U\right]\right.\\\left.\qquad+\,\gamma \left[\left(\frac{1}{{\nu }_{1}}\mathop{\sum }\limits_{i=1}^{{M}_{1}}{J}_{1,i}+\frac{1}{{\nu }_{4}}\mathop{\sum }\limits_{i=1}^{{M}_{4}}{J}_{4,i}\right)-{N}_{3}\right]+\delta \left[\left(\frac{1}{{\nu }_{1}}\mathop{\sum }\limits_{i=1}^{{M}_{1}}{J}_{1,i}+\frac{1}{{\nu }_{3}}\mathop{\sum }\limits_{i=1}^{{M}_{3}}{J}_{3,i}\right)-{N}_{2}\right]\right\}=0\end{array}$$

Thus,

$${J}_{1,i}=-\frac{1}{\frac{\alpha +\gamma +\delta }{{\nu }_{1}}+\beta {\epsilon }_{1,i}},\,{J}_{2,i}=-\frac{1}{-\frac{\alpha }{{\nu }_{2}}+\beta {\epsilon }_{2,i}}$$

$${J}_{3,i}=-\frac{1}{\frac{\delta }{{\nu }_{3}}+\beta {\epsilon }_{3,i}},{J}_{4,i}=-\frac{1}{\frac{\gamma }{{\nu }_{4}}+\beta {\epsilon }_{4,i}}$$

We then introduce the intensive quantities corresponding to the four conservation laws by defining $\beta =1/T$ and $(\alpha +\gamma +\delta )/{\nu }_{1}={\mu }_{1}/T$, $-\alpha /{\nu }_{2}={\mu }_{2}/T$, $\delta /{\nu }_{3}={\mu }_{3}/T$, $\gamma /{\nu }_{4}={\mu }_{4}/T$ where $T$ is the common optical temperature and ${\mu }_{k}$ denotes the corresponding chemical potential. As a result, upon thermalization, for each frequency component, the average power occupancy among the associated spatial modes is found to obey a Rayleigh-Jeans distribution, i.e., Eq. (4), while their chemical potentials ${\mu }_{k}$ are balanced according to Eq. (5).

### Derivation of equations of state

As demonstrated in the main text, at thermal equilibrium, the optical power and internal energy at frequency ${\omega }_{k}$ are given by

7 $${P}_{k}=\mathop{\sum}\limits_{i=1}^{{M}_{k}}{J}_{k,i}$$

8 $${U}_{k}=-\mathop{\sum}\limits_{i=1}^{{M}_{k}}{\epsilon }_{k,i}{J}_{k,i}$$

where each modal occupancy ${J}_{k,i}$ obeys a RJ distribution (Eq. (4)). After substituting the RJ distributions in the above expressions, we obtain

$$\frac{{U}_{k}}{T}-\frac{{\mu }_{k}}{T}{P}_{k}=\mathop{\sum }\limits_{i=1}^{{M}_{k}}\left[\left(\frac{{\epsilon }_{k,i}}{{\epsilon }_{k,i}+{\mu }_{k}}\right)-\left(-\frac{{\mu }_{k}}{{\epsilon }_{k,i}+{\mu }_{k}}\right)\right]=\mathop{\sum }\limits_{i=1}^{{M}_{k}}1={M}_{k}$$

which directly leads to the equations of state

9 $${U}_{k}-{\mu }_{k}{P}_{k}={M}_{k}T$$

### Extensivity of the optical entropy

As indicated in the main text, at equilibrium, the total optical entropy of this multi-frequency multimode system is given by

$$S=\mathop{\sum}\limits_{k}{S}_{k}\left({U}_{k},{M}_{k},{{\mathcal{P}}}_{k}\right)=\mathop{\sum}\limits_{k}\mathop{\sum }\limits_{i}^{{M}_{k}}{\mathrm{ln}}{J}_{k,i}=\mathop{\sum}\limits_{k}\mathop{\sum }\limits_{i}^{{M}_{k}}{\mathrm{ln}}\left(-\frac{T}{{\epsilon }_{k,i}+{\mu }_{k}}\right)$$

where $k$ and $i$ denote the frequency and mode index, respectively. To demonstrate that in these systems, the entropy is indeed extensive, let us now, double for example the number of supermodes ${M}_{k}$ at frequency *ω*_*k*_, i.e., ${M}_{k}\to 2{M}_{k}$ in a self-similar manner. In this respect, each eigenvalue ${\epsilon }_{k,i}$ now splits into two closely spaced “energy” levels ${\epsilon }_{k,i1}$ and ${\epsilon }_{k,i2}$, that approximately satisfy ${\epsilon }_{k,i1}\approx {\epsilon }_{k,i2}\approx {\epsilon }_{k,i}$. At the same time, let us double the optical powers, i.e., *P*_*k*_ → 2*P*_*k*_ in which case *U*_*k*_ → 2*U*_*k*_^43^. From Eq. (9), we find that $T$ and ${\mu }_{k}$ remain the same since $2{U}_{k}-2{\mu }_{k}{P}_{k}=2{M}_{k}T$. As a result, the total entropy now becomes

$${S}^{{\prime} }=\mathop{\sum}\limits_{k}{S}_{k}\left(2{U}_{k},2{M}_{k},2{P}_{k}\right)=\mathop{\sum}\limits_{k}\mathop{\sum }\limits_{i}^{{M}_{k}}\left[{\mathrm{ln}}\left(-\frac{T}{{\epsilon }_{k,i1}+{\mu }_{k}}\right)+{\mathrm{ln}}\left(-\frac{T}{{\epsilon }_{k,i2}+{\mu }_{k}}\right)\right]$$

Hence

$${S}^{{\prime} }=\mathop{\sum}\limits_{k}{S}_{k}\left(2{U}_{k},2{M}_{k},2{P}_{k}\right)=2\mathop{\sum}\limits_{k}\mathop{\sum }\limits_{i}^{{M}_{k}}{\mathrm{ln}}\left(-\frac{T}{{\epsilon }_{k,i}+{\mu }_{k}}\right)=2\mathop{\sum}\limits_{k}{S}_{k}\left({U}_{k},{M}_{k},{P}_{k}\right)=2S$$

This aspect can be further generalized, i.e., ${S}^{{\prime} }={\sum }_{k}{S}_{k}\left(\lambda {U}_{k},\lambda {M}_{k},\lambda {P}_{k}\right)=\lambda {\sum }_{k}{S}_{k}\left({U}_{k},{M}_{k},{P}_{k}\right)=\lambda S$. Thus, the optical entropy as given by Eq. (3) is extensive.

## Supplementary information

Supplementary information: Photon-photon chemical thermodynamics of frequency conversion processes in highly multimode systems
