# Supplementary material for: Photon–photon chemical thermodynamics of frequency conversion processes in highly multimode systems
Source: Light Sci Appl. 2025 May 12;14:188. doi: 10.1038/s41377-025-01856-4 (PMC12069669; doi:10.1038/s41377-025-01856-4)
Supplement: Supplementary file 1 — Supplementary information: Photon-photon chemical thermodynamics of frequency conversion processes in highly multimode systems [file 41377_2025_1856_MOESM1_ESM.docx]

**Supplementary information**

**Photon-photon chemical thermodynamics of frequency conversion processes in highly multimode systems**

**Huizhong Ren^1^, Georgios G. Pyrialakos^1^, Qi Zhong^2^, Fan O. Wu^3^, Mercedeh Khajavikhan^1,4*^, Demetrios N. Christodoulides^1,4*^**

^1^Ming Hsieh Department of Electrical and Computer Engineering, University of Southern California, Los Angeles, CA 90089, USA

^2^CREOL, College of Optics and Photonics, University of Central Florida, Orlando, FL 32816, USA

^3^School of Applied and Engineering Physics, Cornell University, Ithaca, NY 14853, USA

^4^Department of Physics and Astronomy, University of Southern California, Los Angeles, CA 90089, USA

*Corresponding authors: [demetri@usc.edu](mailto:demetri@usc.edu) (D.N.C.), [khajavik@usc.edu](mailto:khajavik@usc.edu) (M.K.)

**A. Conservation laws and field amplitude normalizations for general frequency conversion processes in discrete optical systems**

We here provide the conservation laws and field amplitude normalizations for frequency conversion processes, i.e., $\nu_{1}\omega_{1}+\nu_{2}\omega_{2}\rightleftharpoons\nu_{3}\omega_{3}+\nu_{4}\omega_{4}$, unfolding in a 1-D discrete photonic lattice involving $M$ identical elements. The integers $\nu_{1-4}$ represent the “stoichiometric coefficients” associated with the nonlinear photon conversion. In such a structure, the evolution of the complex optical field amplitude $a_{k,n}$ (unitless) for the frequency $\omega_{k}$ at the $n$th array site is governed by [1]:

$$\begin{aligned} i\frac{da_{k,n}}{dz}+\kappa_{k}\left( a_{k,n+1}+a_{k,n-1} \right)+\frac{\omega_{k}}{4\mathcal{P}_{k}}\iint\vec{e}_{k,n}^{*}\cdot\vec{P}_{NL,k}\left( \vec{r} \right)e^{-i\beta_{k,n}z}dxdy=0\#\left( A.1 \right) \end{aligned}$$

where $\beta_{k,n}$ is the propagation constant at the corresponding site, $\kappa_{k}$ represents the evanescent wave coupling coefficient between neighboring waveguide elements, and $\vec{e}_{k,n}$ and $\mathcal{P}_{k}$ correspond to the real optical mode distribution and the power conveyed in each individual waveguide, respectively. Given that in this work we will assume that all the elements comprising the array are identical, in what follows, we will drop the index $n$ from $\beta_{k,n}$ and $\vec{e}_{k,n}$. Finally, $\vec{P}_{NL,k}\left( \vec{r} \right)$ stands for the nonlinear polarization vector at $\vec{r}=\left( x,y,z \right)$. Note that the above parameters are defined at the optical frequency $\omega_{k}$. In this paper, we assume without any loss of generality that all the frequency conversion processes are type-0, i.e., the frequency reactants and products have the same polarization which allows one to drop the vector notation. For a general frequency conversion process $\nu_{1}\omega_{1}+\nu_{2}\omega_{2}\rightleftharpoons\nu_{3}\omega_{3}+\nu_{4}\omega_{4}$, the nonlinear polarization vector $\bar{P}_{NL}$ is given by

$$\bar{P}_{NL}\left( \vec{r},t \right)=\epsilon_{0}\chi^{\left( m \right)}\bar{E}^{m}\left( \vec{r},t \right)$$

where $m=\nu_{1}+\nu_{2}+\nu_{3}+\nu_{4}-1$ and the coefficient $\chi^{\left( m \right)}$ represents the m-th order susceptibility of the material. In the above expression, the optical field $\bar{E}\left( \vec{r},t \right)$ comprises four distinct frequency components:

$$\bar{E}\left( \vec{r},t \right)=\frac{1}{2}\left[ E_{1}\left( \vec{r} \right)e^{-i\omega_{1}t}+E_{2}\left( \vec{r} \right)e^{-i\omega_{2}t}+E_{3}\left( \vec{r} \right)e^{-i\omega_{3}t}+E_{4}\left( \vec{r} \right)e^{-i\omega_{4}t}+c.c. \right]$$

where $c.c.$ represents the complex conjugate. In this case, the nonlinear polarization is given by

$$\bar{P}_{NL}\left( \vec{r},t \right)=\frac{1}{2^{m}}\epsilon_{0}\chi^{\left( m \right)}\left( E_{1}e^{-i\omega_{1}t}+E_{2}e^{-i\omega_{3}t}+E_{3}e^{-i\omega_{3}t}+E_{4}e^{-i\omega_{4}t}+c.c. \right)^{m}$$

From this last expression, once the stoichiometric condition is imposed ($\nu_{1}\omega_{1}+\nu_{2}\omega_{2}=\nu_{3}\omega_{3}+\nu_{4}\omega_{4}$), the nonlinear real polarization $\bar{P}_{NL,k}\left( \vec{r},t \right)$ component for each frequency species $\omega_{k}$ can be obtained, and can be expressed as usual in terms of its corresponding complex amplitude $P_{NL,k}\left( \vec{r} \right)$, i.e., $\bar{P}_{NL,k}\left( \vec{r},t \right)=\frac{1}{2}\left[ P_{NL,k}\left( \vec{r} \right)e^{-i\omega_{k}t}+c.c. \right]$. In this respect, the complex harmonic components $P_{NL,k}\left( \vec{r} \right)$ of the nonlinear polarization at each frequency are given by:

$$\omega_{1}:P_{NL,1}\left( \vec{r} \right)=\frac{\epsilon_{0}\chi^{\left( m \right)}}{2^{m-1}}\left[ \left( \frac{m!}{\left( \frac{m-1}{2} \right)!\left( \frac{m+1}{2} \right)!}\left| E_{1} \right|^{m-1}+\frac{m!}{\left( \frac{m-1}{2} \right)!\left( \frac{m-1}{2} \right)!}\sum_{j\neq1} \left| E_{j} \right|^{m-1} \right)E_{1}+\frac{m!}{\left( \nu_{1}-1 \right)!\nu_{2}!\nu_{3}!\nu_{4}!}E_{3}^{\nu_{3}}E_{4}^{\nu_{4}}\left( E_{2}^{*} \right)^{\nu_{2}}\left( E_{1}^{*} \right)^{\left( \nu_{1}-1 \right)} \right] (A.2a)$$

$$\omega_{2}: P_{NL,2}\left( \vec{r} \right)=\frac{\epsilon_{0}\chi^{\left( m \right)}}{2^{m-1}}\left[ \left( \frac{m!}{\left( \frac{m-1}{2} \right)!\left( \frac{m+1}{2} \right)!}\left| E_{2} \right|^{m-1}+\frac{m!}{\left( \frac{m-1}{2} \right)!\left( \frac{m-1}{2} \right)!}\sum_{j\neq2} \left| E_{j} \right|^{m-1} \right)E_{2}+\frac{m!}{\nu_{1}!\left( \nu_{2}-1 \right)!\nu_{3}!\nu_{4}!}E_{3}^{\nu_{3}}E_{4}^{\nu_{4}}\left( E_{1}^{*} \right)^{\nu_{1}}\left( E_{2}^{*} \right)^{\left( \nu_{2}-1 \right)} \right] (A.2b)$$

$$\omega_{3}:P_{NL,3}\left( \vec{r} \right)=\frac{\epsilon_{0}\chi^{\left( m \right)}}{2^{m-1}}\left[ \left( \frac{m!}{\left( \frac{m-1}{2} \right)!\left( \frac{m+1}{2} \right)!}\left| E_{3} \right|^{m-1}+\frac{m!}{\left( \frac{m-1}{2} \right)!\left( \frac{m-1}{2} \right)!}\sum_{j\neq3} \left| E_{j} \right|^{m-1} \right)E_{3}+\frac{m!}{\nu_{1}!\nu_{2}!\left( \nu_{3}-1 \right)!\nu_{4}!}E_{1}^{\nu_{1}}E_{2}^{\nu_{2}}\left( E_{4}^{*} \right)^{\nu_{4}}\left( E_{3}^{*} \right)^{\left( \nu_{3}-1 \right)} \right] (A.2c)$$

$$\omega_{4}: P_{NL,4}\left( \vec{r} \right)=\frac{\epsilon_{0}\chi^{\left( m \right)}}{2^{m-1}}\left[ \left( \frac{m!}{\left( \frac{m-1}{2} \right)!\left( \frac{m+1}{2} \right)!}\left| E_{4} \right|^{m-1}+\frac{m!}{\left( \frac{m-1}{2} \right)!\left( \frac{m-1}{2} \right)!}\sum_{j\neq4} \left| E_{j} \right|^{m-1} \right)E_{4}+\frac{m!}{\nu_{1}!\nu_{2}!\nu_{3}!\left( \nu_{4}-1 \right)!}E_{1}^{\nu_{1}}E_{2}^{\nu_{2}}\left( E_{3}^{*} \right)^{\nu_{3}}\left( E_{4}^{*} \right)^{\left( \nu_{4}-1 \right)} \right] (A.2d)$$

Note that the self-phase and cross-phase modulation terms will only appear only when $(m-1)/2$ is an integer which implies that $m$ is an odd number. To exemplify the above results, consider for example, a degenerate four wave-mixing process, i.e., $\omega_{1}+\omega_{2}\rightleftharpoons2\omega_{3}$ where the associated stoichiometric coefficients are $\nu_{1}=\nu_{2}=1, \nu_{3}=2,\nu_{4}=0$ and hence $m=3$, which indicates that this degenerate four-wave mixing conversion is possible in a $\chi^{\left( 3 \right)}$ material. In this case, after substituting the stoichiometric coefficients, the nonlinear polarizations at each frequency are given by:

$$\omega_{1}:P_{NL,1}\left( \vec{r} \right)=\frac{\epsilon_{0}\chi^{\left( 3 \right)}}{4}\left\{ \left[ 3\left| E_{1} \right|^{2}+6\left( \left| E_{2} \right|^{2}+\left| E_{3} \right|^{2} \right) \right]E_{1}+E_{3}^{2}E_{2}^{*} \right\}$$

$$\omega_{2}: P_{NL,2}\left( \vec{r} \right)=\frac{\epsilon_{0}\chi^{\left( 3 \right)}}{4}\left\{ \left[ 3\left| E_{2} \right|^{2}+6\left( \left| E_{1} \right|^{2}+\left| E_{3} \right|^{2} \right) \right]E_{2}+E_{3}^{2}E_{1}^{*} \right\}$$

$$\omega_{3}:P_{NL,3}\left( \vec{r} \right)=\frac{\epsilon_{0}\chi^{\left( 3 \right)}}{4}\left\{ \left[ 3\left| E_{3} \right|^{2}+6\left( \left| E_{1} \right|^{2}+\left| E_{2} \right|^{2} \right) \right]E_{3}+6E_{1}E_{2}E_{3}^{*} \right\},$$

a result that agrees with our analysis. Note that these last three equations correctly provide self-phase and cross-phase modulation effects along with the four-wave mixing products.

We now return to the most general case ($\nu_{1}\omega_{1}+\nu_{2}\omega_{2}=\nu_{3}\omega_{3}+\nu_{4}\omega_{4}$), when a nonlinear discrete waveguide array is involved. By expressing the optical field as a sum of local fields, i.e., $E_{k}\left( \vec{r} \right)=\sum_{n} a_{k,n}e_{k,n}e^{i\beta_{k}z}$ and after substituting this field distribution into the nonlinear polarization Eqs. (A.2) and Eq. (A.1), we obtain

$$i\frac{da_{1,n}}{dz}+\kappa_{1}\left( a_{1,n+1}+a_{1,n-1} \right)+\frac{\omega_{1}}{\mathcal{P}_{1}}Q_{11}\left| a_{1,n} \right|^{m-1}a_{1,n}+\frac{\omega_{1}}{\mathcal{P}_{1}}\sum_{j\neq1} Q_{1j}\left| a_{j,n} \right|^{m-1}a_{1,n}+\nu_{1}Q_{\mathrm{mix}}\frac{\omega_{1}}{\mathcal{P}_{1}}\left( a_{1,n}^{*} \right)^{\left( \nu_{1}-1 \right)}\left( a_{2,n}^{*} \right)^{\nu_{2}}a_{3,n}^{\nu_{3}}a_{4,n}^{\nu_{4}}e^{i\Delta z}=0$$

$$i\frac{da_{2,n}}{dz}+\kappa_{2}\left( a_{2,n+1}+a_{2,n-1} \right)+\frac{\omega_{2}}{\mathcal{P}_{2}}Q_{21}\left| a_{2,n} \right|^{m-1}a_{2,n}+\frac{\omega_{2}}{\mathcal{P}_{2}}\sum_{j\neq2} Q_{2j}\left| a_{j,n} \right|^{m-1}a_{2,n}+\nu_{2}Q_{\mathrm{mix}}\frac{\omega_{2}}{\mathcal{P}_{2}}\left( a_{1,n}^{*} \right)^{\nu_{1}}\left( a_{2,n}^{*} \right)^{\left( \nu_{2}-1 \right)}a_{3,n}^{\nu_{3}}a_{4,n}^{\nu_{4}}e^{i\Delta z}=0$$

$$i\frac{da_{3,n}}{dz}+\kappa_{3}\left( a_{3,n+1}+a_{3,n-1} \right)+\frac{\omega_{3}}{\mathcal{P}_{3}}Q_{31}\left| a_{3,n} \right|^{m-1}a_{3,n}+\frac{\omega_{3}}{\mathcal{P}_{3}}\sum_{j\neq3} Q_{3j}\left| a_{j,n} \right|^{m-1}a_{3,n}+\nu_{3}Q_{\mathrm{mix}}\frac{\omega_{3}}{\mathcal{P}_{3}}a_{1,n}^{\nu_{1}}a_{2,n}^{\nu_{2}}\left( a_{3,n}^{*} \right)^{\left( \nu_{3}-1 \right)}\left( a_{4,n}^{*} \right)^{\nu_{4}}e^{-i\Delta z}=0$$

$$i\frac{da_{4,n}}{dz}+\kappa_{4}\left( a_{4,n+1}+a_{4,n-1} \right)+\frac{\omega_{4}}{\mathcal{P}_{4}}Q_{41}\left| a_{4,n} \right|^{m-1}a_{4,n}+\frac{\omega_{4}}{\mathcal{P}_{4}}\sum_{j\neq4} Q_{4j}\left| a_{j,n} \right|^{m-1}a_{4,n}+\nu_{4}Q_{\mathrm{mix}}\frac{\omega_{4}}{\mathcal{P}_{4}}a_{1,n}^{\nu_{1}}a_{2,n}^{\nu_{2}}\left( a_{3,n}^{*} \right)^{\nu_{3}}\left( a_{4,n}^{*} \right)^{\left( \nu_{4}-1 \right)}e^{-i\Delta z}=0$$

where the $Q$ prefactors are given by $Q_{k1}=\frac{\epsilon_{0}\chi^{\left( m \right)}}{2^{m+1}}\frac{m!}{\left( \frac{m-1}{2} \right)!\left( \frac{m+1}{2} \right)!}\iint e_{k,n}^{m+1}dxdy$, $Q_{kj}=\frac{\epsilon_{0}\chi^{\left( m \right)}}{2^{m+1}}\frac{m!}{\left( \frac{m-1}{2} \right)!\left( \frac{m-1}{2} \right)!}\iint e_{j,n}^{m-1}e_{k,n}^{2}dxdy$ and $Q_{\mathrm{mix}}=$ $\frac{\epsilon_{0}\chi^{\left( m \right)}}{2^{m+1}}\frac{\left( \nu_{1}+\nu_{2}+\nu_{3}+\nu_{4}-1 \right)!}{\nu_{1}!\nu_{2}!\nu_{3}!\nu_{4}!}\iint e_{1,n}^{\nu_{1}}e_{2,n}^{\nu_{2}}e_{3,n}^{\nu_{3}}e_{4,n}^{\nu_{4}}dxdy$ while $\Delta=\nu_{3}\beta_{3}+\nu_{4}\beta_{4}-\nu_{2}\beta_{2}-\nu_{1}\beta_{1}$ denotes the phase-mismatch. Note that, in deriving the above equations, the mode overlap integrals between different waveguide sites have been neglected. Next, by renormalizing the dimensionless field amplitudes $a_{k,n}$ according to $A_{k,n}=\sqrt{\mathcal{P}_{k}/\omega_{k}}a_{k,n}e^{i\beta_{k}z}$, the nonlinear coupled equations above can be reduced to

$$i\frac{dA_{1,n}}{dz}+\beta_{1}A_{1,n}+\kappa_{1}\left( A_{1,n+1}+A_{1,n-1} \right)+f_{11}\left| A_{1,n} \right|^{m-1}A_{1,n}+\sum_{j\neq1} f_{1j}\left| A_{j,n} \right|^{m-1}A_{1,n}+\nu_{1}\Gamma\left( A_{1,n}^{*} \right)^{\left( \nu_{1}-1 \right)}\left( A_{2,n}^{*} \right)^{\nu_{2}}A_{3,n}^{\nu_{3}}A_{4,n}^{\nu_{4}}=0 (A.3a)$$

$$i\frac{dA_{2,n}}{dz}+\beta_{2}A_{2,n}+\kappa_{2}\left( A_{2,n+1}+A_{2,n-1} \right)+f_{21}\left| A_{2,n} \right|^{m-1}A_{2,n}+\sum_{j\neq1} f_{2j}\left| A_{j,n} \right|^{m-1}A_{2,n}+\nu_{2}\Gamma\left( A_{1,n}^{*} \right)^{\nu_{1}}\left( A_{2,n}^{*} \right)^{\left( \nu_{2}-1 \right)}A_{3,n}^{\nu_{3}}A_{4,n}^{\nu_{4}}=0 (A.3b)$$

$$i\frac{dA_{3,n}}{dz}+\beta_{3}A_{3,n}+\kappa_{3}\left( A_{3,n+1}+A_{3,n-1} \right)+f_{31}\left| A_{3,n} \right|^{m-1}A_{3,n}+\sum_{j\neq1} f_{3j}\left| A_{j,n} \right|^{m-1}A_{3,n}+\nu_{3}\Gamma A_{1,n}^{\nu_{1}}A_{2,n}^{\nu_{2}}\left( A_{3,n}^{*} \right)^{\left( \nu_{3}-1 \right)}\left( A_{4,n}^{*} \right)^{\nu_{4}}=0 (A.3c)$$

$$i\frac{dA_{4,n}}{dz}+\beta_{4}A_{4,n}+\kappa_{4}\left( A_{4,n+1}+A_{4,n-1} \right)+f_{41}\left| A_{4,n} \right|^{m-1}A_{4,n}+\sum_{j\neq1} f_{4j}\left| A_{j,n} \right|^{m-1}A_{4,n}+\nu_{4}\Gamma A_{1,n}^{\nu_{1}}A_{2,n}^{\nu_{2}}\left( A_{3,n}^{*} \right)^{\nu_{3}}\left( A_{4,n}^{*} \right)^{\left( \nu_{4}-1 \right)}=0 (A.3d)$$

where $f_{k1}=\left( \sqrt{\frac{\omega_{1}}{\mathcal{P}_{1}}} \right)^{m+1}Q_{k1}$, $f_{kj}=\frac{\omega_{1}}{\mathcal{P}_{1}}\left( \sqrt{\frac{\omega_{j}}{\mathcal{P}_{j}}} \right)^{m-1}Q_{kj}$ and

$\Gamma=Q_{\mathrm{mix}}\sqrt{\left( \frac{\omega_{1}}{\mathcal{P}_{1}} \right)^{\nu_{1}}\left( \frac{\omega_{2}}{\mathcal{P}_{2}} \right)^{\nu_{2}}\left( \frac{\omega_{3}}{\mathcal{P}_{3}} \right)^{\nu_{3}}\left( \frac{\omega_{4}}{\mathcal{P}_{4}} \right)^{\nu_{4}}}$.

Equations (A.3) can be obtained from the following Hamiltonian

$$H=\sum_{k=1}^{4} \sum_{n=1}^{M} \left[ \kappa_{k}\left( A_{k,n}^{*}A_{k,n+1}+A_{k,n}A_{k,n+1}^{*} \right)+\beta_{k}\left| A_{k,n} \right|^{2} \right]+\frac{2}{m+1}\sum_{k=1}^{4} \sum_{n=1}^{M} f_{k1}\left| A_{k,n} \right|^{m+1}+\sum_{k=1}^{4} \sum_{n=1}^{M} \sum_{j\neq k} f_{kj}\left| A_{j,n} \right|^{m-1}\left| A_{k,n} \right|^{2}+\sum_{n=1}^{M} \Gamma\left[ A_{1,n}^{\nu_{1}}A_{2,n}^{\nu_{2}}\left( A_{3,n}^{*} \right)^{\nu_{3}}\left( A_{4,n}^{*} \right)^{\nu_{4}}+\left( A_{1,n}^{*} \right)^{\nu_{1}}\left( A_{2,n}^{*} \right)^{\nu_{2}}A_{3,n}^{\nu_{3}}A_{4,n}^{\nu_{4}} \right] (A.4)$$

In other words, the equations of motion can be derived from Hamiton’s canonical equations:

$$\begin{aligned} \frac{\partial H}{\partial A_{k,n}}=\frac{\partial iA_{k,n}^{*}}{\partial z}\#\left( A.5a \right) \end{aligned}$$

$$\begin{aligned} \frac{\partial H}{\partial iA_{k,n}^{*}}=-\frac{\partial A_{k,n}}{\partial z}\#\left( A.5b \right) \end{aligned}$$

For example, for the frequency component $\omega_{1}$, the Hamiltonian equations yield:

$$\frac{\partial H}{\partial A_{1,n}}=\kappa_{1}\left( A_{1,n-1}^{*}+A_{1,n+1}^{*} \right)+\beta_{1}A_{1,n}^{*}+f_{11}\left| A_{1,n} \right|^{m-1}A_{1,n}^{*}+\sum_{j\neq k} f_{kj}\left| A_{j,n} \right|^{m-1}A_{1,n}^{*}+\nu_{1}\Gamma A_{1,n}^{\nu_{1}-1}A_{2,n}^{\nu_{2}}\left( A_{3,n}^{*} \right)^{\nu_{3}}\left( A_{4,n}^{*} \right)^{\nu_{4}}=\frac{\partial iA_{1,n}^{*}}{\partial z}$$

$$\frac{\partial H}{\partial iA_{1,n}^{*}}=-i\left[ \kappa_{1}\left( A_{1,n+1}+A_{1,n-1} \right)+\beta_{1}A_{1,n}+f_{11}\left| A_{1,n} \right|^{m-1}A_{1,n}+\sum_{j\neq k} f_{kj}\left| A_{j,n} \right|^{m-1}A_{1,n}+\nu_{1}\Gamma\left( A_{1,n}^{*} \right)^{\nu_{1}-1}\left( A_{2,n}^{*} \right)^{\nu_{2}}A_{3,n}^{\nu_{3}}A_{4,n}^{\nu_{4}} \right]=-\frac{\partial A_{1,n}}{\partial z}$$

which are in full agreement with Eq. (A.3a). The equations of motion for the other frequency components can be derived in a similar manner. Being a conservative system, its Hamiltonian is conserved during evolution since it does not depend explicitly on $z$, i.e., $dH/dz=0$:

$$\frac{dH\left( A_{k,n},iA_{k,n}^{*} \right)}{dz}=\sum_{k} \left( \frac{\partial H}{\partial A_{k,n}}\frac{\partial A_{k,n}}{\partial z}+\frac{\partial H}{\partial iA_{k,n}^{*}}\frac{\partial iA_{k,n}^{*}}{\partial z} \right)+\frac{\partial H}{\partial z}$$

$$=\sum_{k} \left( \frac{\partial iA_{k,n}^{*}}{\partial z}\frac{\partial A_{k,n}}{\partial z}-\frac{\partial A_{k,n}}{\partial z}\frac{\partial iA_{k,n}^{*}}{\partial z} \right)+\frac{\partial H}{\partial z}=0$$

In addition, one can formally show that this problem involves three more independent conserved quantities. These are: $N_{1}=P_{1}/\nu_{1}-P_{2}/\nu_{2}$, $N_{2}=P_{1}/\nu_{1}+P_{3}/\nu_{3}$ and $N_{3}=P_{1}/\nu_{1}+P_{4}/\nu_{4}$, where $P_{k}=\sum_{i=1}^{M} \left| c_{k,i} \right|^{2}= \sum_{n=1}^{M} \left| A_{k,n} \right|^{2}$ and $\left| c_{k,i} \right|^{2}$ denotes the modal occupancy of the $i$th supermode at the frequency $\omega_{k}$. Next, we will show that $N_{1}$ is indeed conserved; the conservation of other invariants can be demonstrated in a similar manner.

$$\frac{dN_{1}}{dz}=\sum_{n=1}^{M} \left[ \frac{1}{\nu_{1}}\left( A_{1,n}^{*}\frac{\partial A_{1,n}}{\partial z}+A_{1,n}\frac{\partial A_{1,n}^{*}}{\partial z} \right)-\frac{1}{\nu_{2}}\left( A_{2,n}^{*}\frac{\partial A_{2,n}}{\partial z}+A_{2,n}\frac{\partial A_{2,n}^{*}}{\partial z} \right) \right]$$

$$=\sum_{n=1}^{M} \left\{ \frac{1}{\nu_{1}}\left[ i\nu_{1}\Gamma\left( A_{1,n}^{*} \right)^{\nu_{1}}\left( A_{2,n}^{*} \right)^{\nu_{2}}A_{3,n}^{\nu_{3}}A_{4,n}^{\nu_{4}}+c.c. \right]-\frac{1}{\nu_{2}}\left[ i\nu_{2}\Gamma\left( A_{1,n}^{*} \right)^{\nu_{1}}\left( A_{2,n}^{*} \right)^{\nu_{2}}A_{3,n}^{\nu_{3}}A_{4,n}^{\nu_{4}}+c.c. \right] \right\}$$

$$=0$$

Under weakly nonlinear conditions, the linear Hamiltonian $H_{L}\approx H$ is conserved. In a Hermitian system, $H_{L}$ can be rewritten in a supermode representation using the fact that the super-eigenmodes [2] are orthonormal, hence

$$H_{L}=\sum_{k} \sum_{i=1}^{M} \epsilon_{k,i}\left| c_{k,i} \right|^{2}$$

where $\epsilon_{k,i}$ stands for the propagation eigenvalue of the $i$th spatial supermode at the frequency $\omega_{k}$, which can be obtained by solving the associated linear eigenvalue problem.

In this context, we have demonstrated that $N_{1-3}$ and $H_{L}$ are indeed the invariants associated with a general nonlinear frequency conversion process: $\nu_{1}\omega_{1}+\nu_{2}\omega_{2}\rightleftharpoons\nu_{3}\omega_{3}+\nu_{4}\omega_{4}$.

**B. Conservation laws and field amplitude normalizations for degenerate four-wave mixing in a** $\boldsymbol{\chi}^{\left( \boldsymbol{3} \right)}$ **optical fiber**

In this section, we provide the normalizations and conserved quantities corresponding to a degenerate four-wave mixing process, $2\omega_{1}\rightleftharpoons\omega_{2}+\omega_{3}$, unfolding in a multimode $\chi^{\left( 3 \right)}$ optical fiber when one polarization is involved. The evolution of the optical field $E_{k}\left( \vec{r} \right)$ confined in a refractive index profile $n_{k}\left( \vec{r} \right)$ at frequency $\omega_{k}$ obeys the nonlinear Helmholtz equation:

$$\nabla^{2}E_{k}+\frac{n_{k}^{2}\omega_{k}^{2}}{c^{2}}E_{k}=-\mu_{0}\omega_{k}^{2}P_{NL,k}\left( \vec{r} \right)$$

where $c$ is the speed of light, $\mu_{0}$ denotes the permeability in the vacuum and $P_{NL}$ is again the nonlinear polarization. Following Section A, the stoichiometric coefficients associated with this conversion are $\nu_{1}=2$, $\nu_{3}=\nu_{4}=1$ and $\nu_{2}=0$. In this respect, the nonlinear polarizations for each frequency component are

$$\omega_{1}:P_{NL,1}\left( \vec{r} \right)=\frac{3}{4}\epsilon_{0}\chi^{\left( 3 \right)}\left[ \left| E_{1} \right|^{2}E_{1}+2\left( \left| E_{2} \right|^{2}+\left| E_{3} \right|^{2} \right)E_{1}+2E_{1}^{*}E_{2}E_{3} \right]$$

$$\omega_{2}:P_{NL,2}\left( \vec{r} \right)=\frac{3}{4}\epsilon_{0}\chi^{\left( 3 \right)}\left[ \left| E_{2} \right|^{2}E_{2}+2\left( \left| E_{1} \right|^{2}+\left| E_{3} \right|^{2} \right)E_{2}+E_{1}E_{1}E_{3}^{*} \right]$$

$$\omega_{3}:P_{NL,3}\left( \vec{r} \right)=\frac{3}{4}\epsilon_{0}\chi^{\left( 3 \right)}\left[ \left| E_{3} \right|^{2}E_{3}+2\left( \left| E_{1} \right|^{2}+\left| E_{2} \right|^{2} \right)E_{3}+E_{1}E_{1}E_{2}^{*} \right]$$

Note that in general the refractive index $n_{k}\left( \vec{r} \right)=n_{0}+\Delta n_{k}\left( \vec{r} \right)$ depends on the optical frequency $\omega_{k}$. Here $n_{0}$ denotes the background refractive index at $\omega_{1}$. On the other hand, $\Delta n_{k}\left( \vec{r} \right)$ represents the index profile of the fiber at $\omega_{k}$ after having absorbed the constant frequency deviation of $n_{0}$ at frequencies $\omega_{k}$ when $k\neq1$. Under paraxial conditions (in weakly guiding fibers), the optical field $E_{k}$ can be expressed in terms of a slowly varying field envelope as $E_{k}=U_{k}e^{i\beta_{k}Z}$, where $\beta_{k}=n_{0}\omega_{k}/c$. In this respect, after substituting the nonlinear polarizations, the paraxial wave equation governing the slowly varying electric field envelope $U_{1}$ at frequency $\omega_{1}$ is given by

$$i\frac{\partial U_{1}}{\partial Z}+\frac{1}{2\beta_{1}}\left( \frac{\partial^{2}U_{1}}{\partial X^{2}}+\frac{\partial^{2}U_{1}}{\partial Y^{2}} \right)+\frac{\omega_{1}}{c}\Delta n_{1}U_{1}+\frac{\omega_{1}}{c}n_{2}\mathcal{N}\left( \omega_{1} \right)=0$$

where $n_{2}=3\chi^{\left( 3 \right)}/(8n_{0})$ is the nonlinear coefficient and $\mathcal{N}\left( \omega_{1} \right)=\left| U_{1} \right|^{2}U_{1}+2\left( \left| U_{2} \right|^{2}+\left| U_{3} \right|^{2} \right)U_{1}+2U_{1}^{*}U_{2}U_{3}$ denotes the nonlinear polarization. In the previous equation, $X,Y,Z$ represent actual spatial coordinates. After normalization, i.e., $Z=2\beta_{1}x_{1}^{2}z, X=xx_{1}, Y=yx_{1}$ and $U_{k}=\rho_{k}u_{k}$ where $\rho_{k}=1/\sqrt{2\left( \omega_{k}/c \right)^{2}n_{0}x_{k}^{2}n_{2}}$ we obtain

$$i\frac{du_{1}}{dz}+\left( \frac{\partial^{2}}{\partial x^{2}}+\frac{\partial^{2}}{\partial y^{2}} \right)u_{1}+V_{1}\left( x,y \right)u_{1}\mathcal{+n}\left( \omega_{1} \right)=0$$

where $x_{k}$ represent actual spatial scales (measuring in meters) for each frequency $\omega_{k}$. For the graded-index fiber considered in the main text, $x_{k}$ is given by $x_{k}=\left( \frac{a^{2}c^{2}}{2\omega_{k}^{2}n_{0}^{2}\Delta} \right)^{\frac{1}{4}}$, where $a$ is the fiber core radius and $\Delta$ denotes the relative refractive index contrast [3]. In addition, $V_{1}=2\frac{\omega_{1}^{2}}{c^{2}}n_{0}x_{1}^{2}\left[ n_{1}\left( x,y \right)-n_{0} \right]$ represents the effective optical potential and $\mathcal{n}\left( \omega_{1} \right)$ the corresponding nonlinear polarization, i.e., $\mathcal{n}\left( \omega_{1} \right)=\left| u_{1} \right|^{2}u_{1}+\frac{2}{\rho_{1}^{2}}\left( \rho_{2}^{2}\left| u_{2} \right|^{2}+\rho_{3}^{2}\left| u_{3} \right|^{2} \right)u_{1}+\frac{2\rho_{2}\rho_{3}}{\rho_{1}^{2}}u_{1}^{*}u_{2}u_{3}$. Under weakly nonlinear conditions, the eigenstates (modes) of this optical system $\Phi=\phi_{i}e^{i\epsilon_{i}z}$ at frequency $\omega_{1}$ can be directly obtained by solving the following linear eigenvalue problem:

$$\left( \frac{\partial^{2}}{\partial x^{2}}+\frac{\partial^{2}}{\partial y^{2}} \right)\phi_{i}+V_{1}\left( x,y \right)\phi_{i}=\epsilon_{i}\phi_{i}.$$

The nonlinear evolution equations governing the optical field dynamics of $\omega_{1},\omega_{2},\omega_{3}$in this multimode fiber system are given by

$$i\frac{\partial u_{1}}{\partial z}+\left( \frac{\partial^{2}}{\partial x^{2}}+\frac{\partial^{2}}{\partial y^{2}} \right)u_{1}+V_{1}\left( x,y \right)u_{1}+\left| u_{1} \right|^{2}u_{1}+\frac{2}{\rho_{1}^{2}}\left( \rho_{2}^{2}\left| u_{2} \right|^{2}+\rho_{3}^{2}\left| u_{3} \right|^{2} \right)u_{1}+\frac{2\rho_{2}\rho_{3}}{\rho_{1}^{2}}u_{1}^{*}u_{2}u_{3}=0$$

$$i\frac{\partial u_{2}}{\partial z}+s_{1}^{2}\left( \frac{\partial^{2}}{\partial x^{2}}+\frac{\partial^{2}}{\partial y^{2}} \right)u_{2}+V_{2}\left( x,y \right)u_{2}+\left| u_{2} \right|^{2}u_{2}+\frac{2}{\rho_{2}^{2}}\left( \rho_{1}^{2}\left| u_{1} \right|^{2}+\rho_{3}^{2}\left| u_{3} \right|^{2} \right)u_{2}+\frac{\rho_{1}^{2}\rho_{3}}{\rho_{2}^{3}}u_{1}u_{1}u_{3}^{*}=0$$

$$i\frac{\partial u_{3}}{\partial z}+s_{2}^{2}\left( \frac{\partial^{2}}{\partial x^{2}}+\frac{\partial^{2}}{\partial y^{2}} \right)u_{3}+V_{3}\left( x,y \right)u_{3}+\left| u_{3} \right|^{2}u_{3}+\frac{2}{\rho_{3}^{2}}\left( \rho_{1}^{2}\left| u_{1} \right|^{2}+\rho_{2}^{2}\left| u_{2} \right|^{2} \right)u_{3}+\frac{\rho_{1}^{2}\rho_{2}}{\rho_{3}^{3}}u_{1}u_{1}u_{2}^{*}=0$$

where $s_{1}=x_{1}/x_{2}$ and $s_{2}=x_{1}/x_{3}$. After again renormalizing the field amplitudes according to $A=\sqrt{\frac{\rho_{1}^{2}}{\rho_{2}\rho_{3}}}u_{1}$, $B=\sqrt{\frac{\rho_{2}^{3}}{\rho_{1}^{2}\rho_{3}}}u_{2}$ and $C=\sqrt{\frac{\rho_{3}^{3}}{\rho_{1}^{2}\rho_{2}}}u_{3}$, the equations above are reduced to

$$\begin{aligned} i\frac{\partial A}{\partial z}+\left( \frac{\partial^{2}}{\partial x^{2}}+\frac{\partial^{2}}{\partial y^{2}} \right)A+V_{1}\left( x,y \right)A+\frac{\rho_{2}\rho_{3}}{\rho_{1}^{2}}\left| A \right|^{2}A+2\left( \frac{\rho_{3}}{\rho_{2}}\left| B \right|^{2}+\frac{\rho_{2}}{\rho_{3}}\left| C \right|^{2} \right)A+2A^{*}BC=0\#\left( B.1a \right) \end{aligned}$$

$$\begin{aligned} i\frac{\partial B}{\partial z}+s_{1}^{2}\left( \frac{\partial^{2}}{\partial x^{2}}+\frac{\partial^{2}}{\partial y^{2}} \right)B+V_{2}\left( x,y \right)B+\frac{\rho_{1}^{2}\rho_{3}}{\rho_{2}^{3}}\left| B \right|^{2}B+2\left( \frac{\rho_{3}}{\rho_{2}}\left| A \right|^{2}+\frac{\rho_{1}^{2}}{\rho_{2}\rho_{3}}\left| C \right|^{2} \right)B+A^{2}C^{*}=0\#\left( B.1b \right) \end{aligned}$$

$$\begin{aligned} i\frac{\partial C}{\partial z}+s_{2}^{2}\left( \frac{\partial^{2}}{\partial x^{2}}+\frac{\partial^{2}}{\partial y^{2}} \right)C+V_{3}\left( x,y \right)C+\frac{\rho_{1}^{2}\rho_{2}}{\rho_{3}^{3}}\left| C \right|^{2}C+2\left( \frac{\rho_{2}}{\rho_{3}}\left| A \right|^{2}+\frac{\rho_{1}^{2}}{\rho_{2}\rho_{3}}\left| B \right|^{2} \right)C+A^{2}B^{*}=0\#\left( B.1c \right) \end{aligned}$$

Next, we derive the conserved quantities associated with Eqs. (B.1). The Lagrangian density corresponding to Eqs. (B.1) is given by

$$\mathcal{L=}iA_{z}A^{*}-\left( \left| A_{x} \right|^{2}+\left| A_{y} \right|^{2} \right)+V_{1}\left| A \right|^{2}+\frac{\alpha}{2}\left| A \right|^{4}+2\left( \frac{\rho_{3}}{\rho_{2}}\left| B \right|^{2}+\frac{\rho_{2}}{\rho_{3}}\left| C \right|^{2} \right)\left| A \right|^{2}+A^{2}B^{*}C^{*}$$

$$+iB_{z}B^{*}-s_{1}^{2}\left( \left| B_{x} \right|^{2}+\left| B_{y} \right|^{2} \right)+V_{2}\left| B \right|^{2}+\frac{\beta}{2}\left| B \right|^{4}+\frac{2\rho_{1}^{2}}{\rho_{2}\rho_{3}}\left| B \right|^{2}\left| C \right|^{2}+{A^{*}}^{2}BC$$

$$+iC_{z}C^{*}-s_{2}^{2}\left( \left| C_{x} \right|^{2}+\left| C_{y} \right|^{2} \right)+V_{3}\left| C \right|^{2}+\frac{\gamma}{2}\left| C \right|^{4}$$

where $u_{q}=\frac{\partial u}{\partial q}$ with $u=A,B,C$ and $q=x,y,z$, $\alpha=\frac{\rho_{2}\rho_{3}}{\rho_{1}^{2}}$, $\beta=\frac{\rho_{1}^{2}\rho_{3}}{\rho_{2}^{3}}$ and $\gamma=\frac{\rho_{1}^{2}\rho_{2}}{\rho_{3}^{3}}$. In this respect, Eqs. (B.1) correctly result from the following Euler-Lagrange equations

$$\frac{\partial}{\partial z}\left( \frac{\partial\mathcal{L}}{\partial A_{z}} \right)+\frac{\partial}{\partial x}\left( \frac{\partial\mathcal{L}}{\partial A_{x}} \right)+\frac{\partial}{\partial y}\left( \frac{\partial\mathcal{L}}{\partial A_{y}} \right)-\frac{\partial\mathcal{L}}{\partial A}=0$$

$$\frac{\partial}{\partial z}\left( \frac{\partial\mathcal{L}}{\partial B_{z}} \right)+\frac{\partial}{\partial x}\left( \frac{\partial\mathcal{L}}{\partial B_{x}} \right)+\frac{\partial}{\partial y}\left( \frac{\partial\mathcal{L}}{\partial B_{y}} \right)-\frac{\partial\mathcal{L}}{\partial B}=0$$

$$\frac{\partial}{\partial z}\left( \frac{\partial\mathcal{L}}{\partial C_{z}} \right)+\frac{\partial}{\partial x}\left( \frac{\partial\mathcal{L}}{\partial C_{x}} \right)+\frac{\partial}{\partial y}\left( \frac{\partial\mathcal{L}}{\partial C_{y}} \right)-\frac{\partial\mathcal{L}}{\partial C}=0$$

By introducing the canonical momenta $\pi,\pi^{*},\tau,\tau^{*}, \nu,\nu^{*}$ which are given by $\pi=\frac{\partial\mathcal{L}}{\partial A_{z}}=iA^{*},\pi^{*}=\frac{\partial\mathcal{L}}{\partial A_{z}^{*}}=0,\tau=\frac{\partial\mathcal{L}}{\partial B_{z}}=iB^{*},\tau^{*}=\frac{\partial\mathcal{L}}{\partial B_{z}^{*}}=0,\nu=\frac{\partial\mathcal{L}}{\partial C_{z}}=iC^{*},\nu^{*}=\frac{\partial\mathcal{L}}{\partial C_{z}^{*}}=0$, one can in turn obtain the Hamiltonian density

$$\mathcal{H=}A_{z}\pi+A_{z}^{*}\pi^{*}+B_{z}\tau+B_{z}^{*}\tau^{*}+C_{z}\nu+C_{z}^{*}\nu^{*}\mathcal{-L}$$

$$=\left| A_{x} \right|^{2}+\left| A_{y} \right|^{2}-V_{1}\left| A \right|^{2}-\frac{\alpha}{2}\left| A \right|^{4}+s_{1}^{2}\left( \left| B_{x} \right|^{2}+\left| B_{y} \right|^{2} \right)-V_{2}\left| B \right|^{2}-\frac{\beta}{2}\left| B \right|^{4}+s_{2}^{2}\left( \left| C_{x} \right|^{2}+\left| C_{y} \right|^{2} \right)-V_{3}\left| C \right|^{2}-\frac{\gamma}{2}\left| C \right|^{4}$$

$$-2\left( \frac{\rho_{3}}{\rho_{2}}\left| B \right|^{2}+\frac{\rho_{2}}{\rho_{3}}\left| C \right|^{2} \right)\left| A \right|^{2}-\frac{2\rho_{1}^{2}}{\rho_{2}\rho_{3}}\left| B \right|^{2}\left| C \right|^{2}-A^{2}B^{*}C^{*}-{A^{*}}^{2}BC$$

$$=\mathcal{H}_{A}+\mathcal{H}_{B}+\mathcal{H}_{C}+\mathcal{H}_{int}$$

where

$$\mathcal{H}_{A}=\left| A_{x} \right|^{2}+\left| A_{y} \right|^{2}-V_{1}\left| A \right|^{2}-\frac{\alpha}{2}\left| A \right|^{4}$$

$$\mathcal{H}_{B}=s_{1}^{2}\left( \left| B_{x} \right|^{2}+\left| B_{y} \right|^{2} \right)-V_{2}\left| B \right|^{2}-\frac{\beta}{2}\left| B \right|^{4}$$

$$\mathcal{H}_{C}=s_{2}^{2}\left( \left| C_{x} \right|^{2}+\left| C_{y} \right|^{2} \right)-V_{3}\left| C \right|^{2}-\frac{\gamma}{2}\left| C \right|^{4}$$

$$\mathcal{H}_{int}=-2\left( \frac{\rho_{3}}{\rho_{2}}\left| B \right|^{2}+\frac{\rho_{2}}{\rho_{3}}\left| C \right|^{2} \right)\left| A \right|^{2}-\frac{2\rho_{1}^{2}}{\rho_{2}\rho_{3}}\left| B \right|^{2}\left| C \right|^{2}-A^{2}B^{*}C^{*}-{A^{*}}^{2}BC$$

$$=\frac{2\rho_{3}}{\rho_{2}}AB\pi\tau+\frac{2\rho_{2}}{\rho_{3}}AC\pi\nu+\frac{2\rho_{1}^{2}}{\rho_{2}\rho_{3}}BC\tau\nu+A^{2}\tau\nu+BC\pi^{2}$$

The total Hamiltonian $H\mathcal{=\iint H}dxdy$ can be written as $H=H_{A}+H_{B}+H_{C}+H_{int}$. Note that in the mode representation, the linear component of Hamiltonian can be rewritten as

$$\left\langle H_{L} \right\rangle=-U=\sum_{i=1}^{M_{A}} \epsilon_{i,A}\left| c_{i,A} \right|^{2}+\sum_{i=1}^{M_{B}} \epsilon_{i,B}\left| c_{i,B} \right|^{2}+\sum_{i=1}^{M_{C}} \epsilon_{i,C}\left| c_{i,C} \right|^{2}$$

where $\left| c_{i} \right|^{2}$ are the corresponding modal occupancies. Under weakly nonlinear conditions, the linear Hamiltonian $H_{L}\approx H$ is conserved.

We next identify the pertinent conservation laws that apply for this scenario ($2\omega_{1}=\omega_{2}+\omega_{3}$). To do so, we show that these quantities commute with the Hamiltonian after considering their Poisson bracket. As we will see, these quantities are formally conserved even under nonlinear conditions. We now define the normalized powers:

$$\begin{matrix} N_{A}=\iint\left| A \right|^{2}dxdy, & N_{B}=\iint\left| B \right|^{2}dxdy, & N_{C}=\iint\left| C \right|^{2}dxdy \end{matrix}$$

Let us now consider for example $\left\{ N_{A},H \right\}$. In this case, the Poisson bracket $\left\{ N_{A},H \right\}=\left\{ N_{A},H_{A}+H_{B}+H_{C}+H_{int} \right\}=\left\{ N_{A},H_{A} \right\}+\left\{ N_{A},H_{B}+H_{c} \right\}+\left\{ N_{A},H_{int} \right\}$. The first term can be calculated by substituting $\mathcal{N}_{A}=\left| A \right|^{2}=-iA\pi$ and $\mathcal{H}_{A}=-i\left( A_{x}\pi_{x}+A_{y}\pi_{y} \right)+iV_{1}A\pi+\frac{\alpha}{2}\left( A\pi\right)^{2}$ into the following integral:

$$\left\{ N_{A},H_{A} \right\}=\iint\left( \frac{\delta\mathcal{N}_{A}}{\delta A}\frac{\delta\mathcal{H}_{A}}{\delta\pi}-\frac{\delta\mathcal{H}_{A}}{\delta A}\frac{\delta\mathcal{N}_{A}}{\delta\pi} \right)dxdy$$

where

$$\frac{\delta\mathcal{N}_{A}}{\delta A}=\frac{\partial\mathcal{N}_{A}}{\partial A}-\partial_{x}\left( \frac{\partial\mathcal{N}_{A}}{\partial A_{x}} \right)-\partial_{y}\left( \frac{\partial\mathcal{N}_{A}}{\partial A_{y}} \right)=\frac{\partial\mathcal{N}_{A}}{\partial A}=-i\pi$$

$$\frac{\delta\mathcal{N}_{A}}{\delta\pi}=\frac{\partial\mathcal{N}_{A}}{\partial\pi}-\partial_{x}\left( \frac{\partial\mathcal{N}_{A}}{\partial\pi_{x}} \right)-\partial_{y}\left( \frac{\partial\mathcal{N}_{A}}{\partial\pi_{y}} \right)=\frac{\partial\mathcal{N}_{A}}{\partial\pi}=-iA$$

$$\frac{\delta\mathcal{H}_{A}}{\delta A}=\frac{\partial\mathcal{H}_{A}}{\partial A}-\partial_{x}\left( \frac{\partial\mathcal{H}_{A}}{\partial A_{x}} \right)-\partial_{y}\left( \frac{\partial\mathcal{H}_{A}}{\partial A_{y}} \right)=iV_{1}\pi+\alpha A\pi^{2}+i\left( \pi_{xx}+\pi_{yy} \right)$$

$$\frac{\delta\mathcal{H}_{A}}{\delta\pi}=\frac{\partial\mathcal{H}_{A}}{\partial\pi}-\partial_{x}\left( \frac{\partial\mathcal{H}_{A}}{\partial\pi_{x}} \right)-\partial_{y}\left( \frac{\partial\mathcal{H}_{A}}{\partial\pi_{y}} \right)=iV_{1}A+\alpha A^{2}\pi+i\left( A_{xx}+A_{yy} \right)$$

Thus

$$\left\{ N_{A},H_{A} \right\}=\iint\left\{ -i\pi\left[ iV_{1}A+\alpha A^{2}\pi+i\left( A_{xx}+A_{yy} \right) \right]+iA\left[ iV_{1}\pi+\alpha A\pi^{2}+i\left( \pi_{xx}+\pi_{yy} \right) \right] \right\}dxdy$$

$$=\iint\left[ \pi\left( A_{xx}+A_{yy} \right)-A\left( \pi_{xx}+\pi_{yy} \right) \right]dxdy=i\iint\left[ A^{*}\left( A_{xx}+A_{yy} \right)-A\left( A_{xx}^{*}+A_{yy}^{*} \right) \right]dxdy$$

$$=i\iint\left[ \partial_{x}\left( A^{*}A_{x}-AA_{x}^{*} \right)+\partial_{y}\left( A^{*}A_{y}-AA_{y}^{*} \right) \right]dxdy$$

$$=i[\int dy\left. \left( A^{*}A_{x}-AA_{x}^{*} \right) \right|_{-\infty}^{+\infty}+\int dx\left. \left( A^{*}A_{y}-AA_{y}^{*} \right) \right|_{-\infty}^{+\infty}=0$$

Note that the above integral is zero since the guided mode fields vanish at infinity. Hence, $\left\{ N_{A},H_{A} \right\}=0$. The second term is also zero since $\mathcal{N}_{A}=\mathcal{N}_{A}\left( A,\pi\right)$ and $\mathcal{H}_{B}+\mathcal{H}_{C}=\mathcal{H}_{B}\left( B,\tau\right)+\mathcal{H}_{C}\left( C,\nu\right)$. Finally, the last term can be calculated as follows: after substituting $\mathcal{N}_{A}=\left| A \right|^{2}=-iA\pi$ and $\mathcal{H}_{int}=\frac{2\rho_{3}}{\rho_{2}}AB\pi\tau+\frac{2\rho_{2}}{\rho_{3}}AC\pi\nu+\frac{2\rho_{1}^{2}}{\rho_{2}\rho_{3}}BC\tau\nu+A^{2}\tau\nu+BC\pi^{2}$ into the integral, we obtain

$$\left\{ N_{A},H_{int} \right\}=\iint\left( \frac{\delta\mathcal{N}_{A}}{\delta A}\frac{\delta\mathcal{H}_{int}}{\delta\pi}-\frac{\delta\mathcal{H}_{int}}{\delta A}\frac{\delta\mathcal{N}_{A}}{\delta\pi}+\frac{\delta\mathcal{N}_{A}}{\delta B}\frac{\delta\mathcal{H}_{int}}{\delta\tau}-\frac{\delta\mathcal{H}_{int}}{\delta B}\frac{\delta\mathcal{N}_{A}}{\delta\tau}+\frac{\delta\mathcal{N}_{A}}{\delta C}\frac{\delta\mathcal{H}_{int}}{\delta\nu}-\frac{\delta\mathcal{H}_{int}}{\delta C}\frac{\delta\mathcal{N}_{A}}{\delta\nu} \right)dxdy$$

$$=\iint\left( \frac{\delta\mathcal{N}_{A}}{\delta A}\frac{\delta\mathcal{H}_{int}}{\delta\pi}-\frac{\delta\mathcal{H}_{int}}{\delta A}\frac{\delta\mathcal{N}_{A}}{\delta\pi} \right)dxdy$$

where

$$\frac{\delta\mathcal{H}_{int}}{\delta A}=\frac{\partial\mathcal{H}_{int}}{\partial A}-\partial_{x}\left( \frac{\partial\mathcal{H}_{int}}{\partial A_{x}} \right)-\partial_{y}\left( \frac{\partial\mathcal{H}_{int}}{\partial A_{y}} \right)=\frac{\partial\mathcal{H}_{int}}{\partial A}$$

$$\frac{\delta\mathcal{H}_{int}}{\delta\pi}=\frac{\partial\mathcal{H}_{int}}{\partial\pi}-\partial_{x}\left( \frac{\partial\mathcal{H}_{int}}{\partial\pi_{x}} \right)-\partial_{y}\left( \frac{\partial\mathcal{H}_{int}}{\partial\pi_{y}} \right)=\frac{\partial\mathcal{H}_{int}}{\partial\pi}$$

Hence

$$\left\{ N_{A},H_{int} \right\}=\iint\left[ -i\pi\left( \frac{2\rho_{3}}{\rho_{2}}AB\tau+\frac{2\rho_{2}}{\rho_{3}}AC\nu+2\pi BC \right)+iA\left( \frac{2\rho_{3}}{\rho_{2}}B\pi\tau+\frac{2\rho_{2}}{\rho_{3}}C\pi\nu+2A\tau\nu\right) \right]dxdy$$

$$=\iint\left( -2i\pi^{2}BC+2iA^{2}\tau\nu\right)dxdy$$

Similarly, one can obtain the Poisson bracket of $\left\{ N_{B},H \right\}$ and $\left\{ N_{C},H \right\}$. Following a similar procedure, we can show that $\left\{ N_{B},H \right\}=\left\{ N_{B},H_{A}+H_{B}+H_{C}+H_{int} \right\}=\left\{ N_{B},H_{int} \right\}$ and $\left\{ N_{C},H \right\}=\left\{ N_{C},H_{A}+H_{B}+H_{C}+H_{int} \right\}=\left\{ N_{C},H_{int} \right\}$ where $\mathcal{N}_{B}=\left| B \right|^{2}=-iB\tau, \mathcal{N}_{C}=\left| C \right|^{2}=-iC\nu$. After substituting the power density and the nonlinear interaction Hamiltonian into the integral, we obtain

$$\left\{ N_{B},H_{int} \right\}=\iint\left( \frac{\delta\mathcal{N}_{B}}{\delta B}\frac{\delta\mathcal{H}_{int}}{\delta\tau}-\frac{\delta\mathcal{H}_{int}}{\delta B}\frac{\delta\mathcal{N}_{B}}{\delta\tau} \right)dxdy$$

$$=\iint\left[ -i\tau\left( \frac{2\rho_{3}}{\rho_{2}}AB\pi+\frac{2\rho_{1}^{2}}{\rho_{2}\rho_{3}}BC\nu+A^{2}\nu\right)+iB\left( \frac{2\rho_{3}}{\rho_{2}}A\pi\tau+\frac{2\rho_{1}^{2}}{\rho_{2}\rho_{3}}C\tau\nu+\pi^{2}C \right) \right]dxdy$$

$$=\iint\left( -iA^{2}\tau\nu+i\pi^{2}BC \right)dxdy$$

$$\left\{ N_{C},H_{int} \right\}=\iint\left( \frac{\delta\mathcal{N}_{C}}{\delta C}\frac{\delta\mathcal{H}_{int}}{\delta\nu}-\frac{\delta\mathcal{H}_{int}}{\delta C}\frac{\delta\mathcal{N}_{C}}{\delta\nu} \right)dxdy$$

$$=\iint\left[ -i\nu\left( \frac{2\rho_{2}}{\rho_{3}}AC\pi+\frac{2\rho_{1}^{2}}{\rho_{2}\rho_{3}}BC\tau+A^{2}\tau\right)+iC\left( \frac{2\rho_{2}}{\rho_{3}}A\pi\nu+\frac{2\rho_{1}^{2}}{\rho_{2}\rho_{3}}B\tau\nu+B\pi^{2} \right) \right]dxdy$$

$$=\iint\left( -iA^{2}\tau\nu+i\pi^{2}BC \right)dxdy$$

Thus, one can formally show that indeed

$$\begin{matrix} \left\{ \frac{N_{A}}{2}+N_{B},H \right\}=0, & \left\{ N_{B}-N_{C},H \right\}=0 \end{matrix}$$

Therefore, the quantities $\frac{N_{A}}{2}+N_{B}$, $N_{B}-N_{C}$ are conserved. Meanwhile, under weak nonlinear conditions, the internal energy $U=-\left\langle H_{L} \right\rangle$ is also to a good approximation conserved (quasi-conserved).

**C. Conservation laws for second-harmonic generation in a** $\boldsymbol{\chi}^{\left( \boldsymbol{2} \right)}$ **LiNbO_3_ waveguide lattice**

In this section, we provide the conserved quantities and normalizations associated with second-harmonic generation (SHG) taking place in a $\chi^{\left( 2 \right)}$ LiNbO_3_ waveguide lattice, as discussed in the main text. According to Section A, the stoichiometric coefficients associated with SHG $2\omega_{A}\rightleftharpoons\omega_{B}$ are $\nu_{1}=2$, $\nu_{3}=1$ and $\nu_{2}=\nu_{4}=0$. In this respect, the governing equations can be obtained from Eqs. (A.3):

$$\begin{aligned} i\frac{dA_{n}}{dz}+\beta_{A}A_{n}+\kappa_{A}\left( A_{n+1}+A_{n-1} \right)+2\Gamma A_{n}^{*}B_{n}=0\#\left( C.1a \right) \end{aligned}$$

$$\begin{aligned} i\frac{dB_{n}}{dz}+\beta_{B}B_{n}+\kappa_{B}\left( B_{n+1}+B_{n-1} \right)+\Gamma A_{n}^{2}=0\#\left( C.1b \right) \end{aligned}$$

where $A_{n}$ and $B_{n}$ are the normalized field amplitudes (in units of $\sqrt{\mathrm{Joule}}$) corresponding to the fundamental frequency $\omega_{A}$ and the second harmonic $\omega_{B}$, respectively. The nonlinear coefficient $\Gamma$ (in units of [$m^{-1}\times\sqrt{\mathrm{Joule}^{-1}}$]) is given by $\Gamma=\frac{\epsilon_{0}\chi^{\left( 2 \right)}}{8}\sqrt{\left( \frac{\omega_{A}}{\mathcal{P}_{A}} \right)^{2}\frac{\omega_{B}}{\mathcal{P}_{B}}}\iint e_{A,n}^{2}e_{B,n}dxdy$.

If we normalize the propagation distance $Z=\kappa_{A}z$, and the optical fields according to $U_{n}=\frac{\Gamma A_{n}}{\kappa_{A}}e^{-i\beta_{A}z}$ and $V_{n}=\frac{\Gamma B_{n}}{\kappa_{A}}e^{-i2\beta_{A}z}$, we obtain

$$\begin{aligned} i\frac{dU_{n}}{dZ}+U_{n+1}+U_{n-1}+2U_{n}^{*}V_{n}=0\#\left( C.2a \right) \end{aligned}$$

$$\begin{aligned} i\frac{dV_{n}}{dZ}+\frac{\kappa_{B}}{\kappa_{A}}\left( V_{n+1}+V_{n-1} \right)+\Delta V_{n}+U_{n}^{2}=0\#\left( C.2b \right) \end{aligned}$$

where $\Delta=(\beta_{B}-2\beta_{A})/\kappa_{A}$ denotes the phase mismatch.

In this case, the two conserved quantities are given by

$$N_{1}=\frac{P_{A}}{2}+P_{B}=\sum_{n=1}^{M} \left( \frac{\left| U_{n} \right|^{2}}{2}+\left| V_{n} \right|^{2} \right)=\sum_{i=1}^{M} \left( \frac{\left| c_{A,i} \right|^{2}}{2}+\left| c_{B,i} \right|^{2} \right)$$

$$U=-\sum_{i=1}^{M} \left( \epsilon_{A,i}\left| c_{A,i} \right|^{2}+\epsilon_{B,i}\left| c_{B,i} \right|^{2} \right)$$

where the last one is applicable under weakly nonlinear conditions. Next, we derive the actual power conveyed in this waveguide array arrangement. As mentioned above, the normalized power for the fundamental frequency $\omega_{A}$ is given by $P_{A}=\sum_{n=1}^{M} \left| U_{n} \right|^{2}=\frac{\Gamma^{2}}{\kappa_{A}^{2}}\sum_{n=1}^{M} \left| A_{n} \right|^{2}$, where $A_{n}=a_{A,n}\sqrt{\mathcal{P}_{A}/\omega_{A}}e^{i\beta_{A}z}$. The actual power conveyed in the waveguide system (in physical units, Watt) is

$$\tilde{P}_{A}=\frac{cn_{0}\epsilon_{0}}{2}\iint\left| E \right|^{2}dxdy=\frac{cn_{0}\epsilon_{0}}{2}\sum_{n} \left| a_{A,n} \right|^{2}\iint\left| e_{A}\left( x,y \right) \right|^{2}dxdy=\mathcal{P}_{A}\sum_{n} \left| a_{A,n} \right|^{2}$$

where $c$ is the speed of light, $\epsilon_{0}$ denotes the vacuum permittivity, $n_{0}$ represents the material refractive index and $\mathcal{P}_{A}$ stands for the optical power conveyed in each local mode at the fundamental frequency. Thus, the actual power is given by

$$\tilde{P}_{A}=\mathcal{P}_{A}\sum_{n} \left| a_{A,n} \right|^{2}=\mathcal{P}_{A}\sum_{n} \left| A_{n} \right|^{2}\times\frac{\omega_{A}}{\mathcal{P}_{A}}=\frac{\omega_{A}P_{A}\kappa_{A}^{2}}{\Gamma^{2}}$$

For the second-harmonic generation discussed in the main text, the normalized input power is $P_{A}=1.5$ at the fundamental wavelength of $\lambda_{A}=1550 \mathrm{nm}$ (see Section F), and the coupling coefficient is $\kappa_{A}=104.7 m^{-1}$. The coefficient $\Gamma$ is directly calculated from the overlap integral of the modes as obtained from COMSOL simulations. The nonlinear susceptibility for lithium niobate is here taken to be $\chi^{\left( 2 \right)}\approx30\times{10}^{-12} \mathrm{mV}^{-1}$. Consequently, the nonlinear coefficient $\Gamma$ is found to be $1.34\times{10}^{9}$ $m^{-1}\times\sqrt{\mathrm{Joule}^{-1}}$. For a normalized total power in the array of $P_{A}=1.5$ at the fundamental wavelength, this will correspond to an actual power of 12 Watts in this 30-element waveguide array.

**D. Conservation laws for sum-frequency generation in a** $\boldsymbol{\chi}^{\left( \boldsymbol{2} \right)}$ **LiNbO_3_ lattice**

In this section, we provide the conserved quantities (Manely-Rowe relations) and normalizations when dealing with sum-frequency generation (SFG) taking place in a $\chi^{\left( 2 \right)}$ LiNbO3 waveguide lattice, as discussed in the main text. According to Supplementary A, the stoichiometric coefficients for SFG $\omega_{A}+\omega_{B}\rightleftharpoons\omega_{C}$ are $\nu_{1}=\nu_{2}=\nu_{3}=1$, and $\nu_{4}=0$. In this respect, the evolution equations can be obtained from Eqs. (A.3):

$$\begin{aligned} i\frac{dA_{n}}{dz}+\beta_{A}A_{n}+\kappa_{A}\left( A_{n+1}+A_{n-1} \right)+\Gamma B_{n}^{*}C_{n}=0\#\left( D.1a \right) \end{aligned}$$

$$\begin{aligned} i\frac{dB_{n}}{dz}+\beta_{B}B_{n}+\kappa_{B}\left( B_{n+1}+B_{n-1} \right)+\Gamma A_{n}^{*}C_{n}=0\#\left( D.1b \right) \end{aligned}$$

$$\begin{aligned} i\frac{dC_{n}}{dz}+\beta_{C}C_{n}+\kappa_{C}\left( C_{n+1}+C_{n-1} \right)+\Gamma A_{n}B_{n}=0\#\left( D.1c \right) \end{aligned}$$

where $A_{n},B_{n}$ and $C_{n}$ represent the normalized field amplitudes (in units of $\sqrt{\mathrm{Joule}}$) corresponding to frequencies $\omega_{A},\omega_{B}$ and $\omega_{C}$, respectively. In addition, the nonlinear coefficient (in units of [$m^{-1}\times\sqrt{\mathrm{Joule}^{-1}}$]) is given by $\Gamma=$ $\frac{\epsilon_{0}\chi^{\left( 2 \right)}}{4}\sqrt{\frac{\omega_{A}\omega_{B}\omega_{C}}{\mathcal{P}_{A}\mathcal{P}_{B}\mathcal{P}_{C}}}\iint e_{A,n}e_{B,n}e_{C,n}dxdy$.

If we let again $Z=\kappa_{A}z$, $U_{n}=\frac{\Gamma A_{n}}{\kappa_{A}}e^{-i\beta_{A}z}$, $V_{n}=\frac{\Gamma B_{n}}{\kappa_{A}}e^{-i\beta_{B}z}$ and $W_{n}=\frac{\Gamma C_{n}}{\kappa_{A}}e^{-i\left( \beta_{A}+\beta_{B} \right)z}$, we obtain

$$\begin{aligned} i\frac{dU_{n}}{dZ}+U_{n+1}+U_{n-1}+V_{n}^{*}W_{n}=0\#\left( D.2a \right) \end{aligned}$$

$$\begin{aligned} i\frac{dV_{n}}{dZ}+\frac{\kappa_{B}}{\kappa_{A}}\left( V_{n+1}+V_{n-1} \right)+U_{n}^{*}W_{n}=0\#\left( D.2b \right) \end{aligned}$$

$$\begin{aligned} i\frac{dW_{n}}{dZ}+\Delta W_{n}+\frac{\kappa_{C}}{\kappa_{A}}\left( W_{n+1}+W_{n-1} \right)+U_{n}V_{n}=0\#\left( D.2c \right) \end{aligned}$$

where $\Delta=(\beta_{C}-\beta_{A}-\beta_{B})/\kappa_{A}$ is the phase mismatch term.

The conserved quantities associated with Eqs. (D.2) are

$$N_{1}=P_{A}-P_{B}=\sum_{n=1}^{M} \left( \left| U_{n} \right|^{2}-\left| V_{n} \right|^{2} \right)=\sum_{i=1}^{M} \left( \left| c_{A,i} \right|^{2}-\left| c_{B,i} \right|^{2} \right)$$

$$N_{2}=P_{A}+P_{C}=\sum_{n=1}^{M} \left( \left| U_{n} \right|^{2}+\left| W_{n} \right|^{2} \right)=\sum_{i=1}^{M} \left( \left| c_{A,i} \right|^{2}+\left| c_{C,i} \right|^{2} \right)$$

$$U=-\sum_{i=1}^{M} \left( \epsilon_{A,i}\left| c_{A,i} \right|^{2}+\epsilon_{B,i}\left| c_{B,i} \right|^{2}+\epsilon_{C,i}\left| c_{C,i} \right|^{2} \right)$$

where again the conservation of the internal energy $U$ is assumed under weakly nonlinear conditions. Note that the dynamics described by Eqs. (D.2) are identical to those of Eqs. (D.1). Therefore, when analyzing the effects of phase mismatch on the frequency conversion efficiency, we can focus solely on the eigenvalue spectrum shift of the frequency component $\omega_{C}$.

We then derive the actual power conveyed in the waveguide array arrangements. As mentioned above, the normalized powers for the reactant frequency components are given by $P_{A}=\sum_{n=1}^{M} \left| U_{n} \right|^{2}=\frac{\Gamma^{2}}{\kappa_{A}^{2}}\sum_{n=1}^{M} \left| A_{n} \right|^{2}$ and $P_{B}=\sum_{n=1}^{M} \left| V_{n} \right|^{2}=\frac{\Gamma^{2}}{\kappa_{A}^{2}}\sum_{n=1}^{M} \left| B_{n} \right|^{2}$, respectively, where $A_{n}=a_{A,n}\sqrt{\mathcal{P}_{A}/\omega_{A}}e^{i\beta_{A}z}$ and $B_{n}=a_{B,n}\sqrt{\mathcal{P}_{B}/\omega_{B}}e^{i\beta_{B}z}$. The actual powers conveyed in the waveguide system (in physical units of Watts) corresponding to the frequencies $\omega_{A}$ and $\omega_{B}$ are

$$\begin{matrix} \tilde{P}_{A}=\mathcal{P}_{A}\sum_{n} \left| a_{A,n} \right|^{2}, & \tilde{P}_{B}=\mathcal{P}_{B}\sum_{n} \left| a_{B,n} \right|^{2} \end{matrix}$$

respectively, where $\mathcal{P}_{A},\mathcal{P}_{B}$ stand for the optical power conveyed in each local mode at $\omega_{A}$ and $\omega_{B}$. Thus, the actual powers flowing in the array system are given by

$$\begin{matrix} \tilde{P}_{A}=\mathcal{P}_{A}\sum_{n} \left| a_{A,n} \right|^{2}=\mathcal{P}_{A}\sum_{n} \left| A_{n} \right|^{2}\times\frac{\omega_{A}}{\mathcal{P}_{A}}=\frac{\omega_{A}P_{A}\kappa_{A}^{2}}{\Gamma^{2}} \\ \tilde{P}_{B}=\mathcal{P}_{B}\sum_{n} \left| a_{B,n} \right|^{2}=\mathcal{P}_{B}\sum_{n} \left| B_{n} \right|^{2}\times\frac{\omega_{B}}{\mathcal{P}_{B}}=\frac{\omega_{B}P_{B}\kappa_{A}^{2}}{\Gamma^{2}} \end{matrix}$$

For the sum-frequency generation discussed in the main text, the normalized input powers are $P_{A}=P_{B}=1$ at wavelengths $\lambda_{A}=1500 \mathrm{nm}$ and $\lambda_{B}=1300 \mathrm{nm}$ (see Section G), while the coupling coefficient is given by $\kappa_{A}=234.6 m^{-1}$. The coefficient $\Gamma$ is directly calculated from the overlap integral of the modes as obtained from COMSOL simulations. Meanwhile the nonlinear susceptibility for lithium niobate is $\chi^{\left( 2 \right)}\approx30\times{10}^{-12} \mathrm{mV}^{-1}$. In this respect, the nonlinear coefficient $\Gamma$ is approximately $4.5\times{10}^{9} m^{-1}\times\sqrt{\mathrm{Joule}^{-1}}$. For a normalized total power in the array of $P_{A}=P_{B}=1$ at frequency $\omega_{A}$ and $\omega_{B}$, these will correspond to an actual power of $\tilde{P}_{A}=3.4 W$ and $\tilde{P}_{B}=4 W$ in this 30-element waveguide array.

**E. Theoretical methodology for predicting the final optical temperature and chemical potentials**

The global thermal equilibrium temperature $T$ that applies to all frequency species and the chemical potentials $\mu_{k}$ for each frequency component, can be formally predicted from the initial excitation conditions ($P_{k},U_{k}$), the equations of state, Eq. (6), and the chemical potential stoichiometric relation of Eq. (5). For example, in a general frequency conversion process described by $\nu_{1}\omega_{1}+\nu_{2}\omega_{2}\rightleftharpoons\nu_{3}\omega_{3}+\nu_{4}\omega_{4}$, we have shown that the following quantities are conserved:

$$N_{1}=\frac{P_{1}}{\nu_{1}}-\frac{P_{2}}{\nu_{2}}$$

$$N_{2}=\frac{P_{1}}{\nu_{1}}+\frac{P_{3}}{\nu_{3}}$$

$$N_{3}=\frac{P_{1}}{\nu_{1}}+\frac{P_{4}}{\nu_{4}}$$

$$U=U_{1}+U_{2}+U_{3}+U_{4}$$

At thermal equilibrium, the power occupancies follow the Rayleigh-Jeans distributions of Eq. (4), while the chemical potentials are balanced through

$$\nu_{1}\mu_{2}+\nu_{2}\mu_{2}=\nu_{3}\mu_{3}+\nu_{4}\mu_{4}$$

After substituting the Rayleigh-Jeans distributions and the equations of state, we obtain

$$\frac{1}{\nu_{1}}\sum_{i=1}^{M_{1}} \left( -\frac{T}{\epsilon_{1,i}+\mu_{1}} \right)-\frac{1}{\nu_{2}}\sum_{i=1}^{M_{2}} \left( -\frac{T}{\epsilon_{2,i}+\mu_{2}} \right)=\frac{P_{1}}{\nu_{1}}-\frac{P_{2}}{\nu_{2}}$$

$$\frac{1}{\nu_{1}}\sum_{i=1}^{M_{1}} \left( -\frac{T}{\epsilon_{1,i}+\mu_{1}} \right)+\frac{1}{\nu_{3}}\sum_{i=1}^{M_{3}} \left( -\frac{T}{\epsilon_{3,i}+\mu_{3}} \right)=\frac{P_{1}}{\nu_{1}}+\frac{P_{3}}{\nu_{3}}$$

$$\frac{1}{\nu_{1}}\sum_{i=1}^{M_{1}} \left( -\frac{T}{\epsilon_{1,i}+\mu_{1}} \right)+\frac{1}{\nu_{4}}\sum_{i=1}^{M_{4}} \left( -\frac{T}{\epsilon_{4,i}+\mu_{4}} \right)=\frac{P_{1}}{\nu_{1}}+\frac{P_{4}}{\nu_{4}}$$

$$\sum_{k=1}^{4} M_{k}T+\sum_{i=1}^{M_{1}} \left( -\frac{\mu_{1}T}{\epsilon_{1,i}+\mu_{1}} \right)+\sum_{i=1}^{M_{2}} \left( -\frac{\mu_{2}T}{\epsilon_{2,i}+\mu_{2}} \right)+\sum_{i=1}^{M_{3}} \left( -\frac{\mu_{3}T}{\epsilon_{1,i}+\mu_{1}} \right)+\sum_{i=1}^{M_{4}} \left( -\frac{\mu_{4}T}{\epsilon_{4,i}+\mu_{4}} \right)=U$$

The five unknown quantities, $\mu_{1-4}$ and $T$, can then be numerically determined from the above five equations.

**F. Waveguide design for second-harmonic generation**

We here present a possible optical waveguide array design to facilitate type-0 second-harmonic generation, as discussed in the main text. The array consists of 30 parallel channel waveguides, equally spaced at a distance of 15 $\mu m$, fabricated via titanium in-diffusion into a periodically poled lithium niobate crystal [4-7]. The diffusion width and depth are approximately 10 $\mu m$ and 5 $\mu m$, respectively. The graded refractive index change from titanium in-diffusion is assumed to be $\Delta n=5\times{10}^{-3}$. As shown in Fig. S1, these channels support a single transverse magnetic (TM) mode for the fundamental wave (FW) at $1550 nm$, while supporting four modes for the second-harmonic wave (SH). The mode profiles were calculated using finite element methods. Due to modal symmetry, the FW TM_00_ mode cannot phase match with the SH TM_01_ mode, as their field overlap integral is zero. To achieve significant harmonic wave coupling, quasi-phase matching via periodic poling is employed to phase-match the fundamental TM_00_ mode with the second harmonic TM_02_ mode. The phase mismatch between these modes is calculated as $\Delta k=\frac{2\pi n_{2}}{\lambda_{2}}-2\times\frac{2\pi n_{1}}{\lambda_{1}}=3.3\times{10}^{5} m^{-1}$, resulting into a required poling period of $\Lambda=\frac{2\pi}{\Delta k}=18.9 \mu m$. The small phase mismatch can be further fine-tuned by adjusting the sample temperature $T$ [7].

Next, we calculate the coupling coefficients associated with the FW TM_00_ and SH TM_02_ mode. Figure S2 illustrates the coupled odd and even modes for FW and SH associated with a pair of such lithium niobate waveguides. The effective refractive indices of the FW even and odd modes are 2.13844 and 2.13839 respectively, while for the SH are 2.179399 and 2.179375. In this respect, the coupling coefficient can be obtained via $\kappa=(2\pi/\lambda)(n_{\mathrm{even}}-n_{\mathrm{odd}})/2$. Thus, the coupling coefficients for FW and SH are given by $\kappa\left( \mathrm{FW} \right)\approx104.7 m^{-1}$, $\kappa\left( \mathrm{SH} \right)\approx97.3 m^{-1}$, the coupling lengths are approximately $L\left( \mathrm{FW} \right)=15 \mathrm{mm}$ and $L\left( \mathrm{SH} \right)=16 \mathrm{mm}$. In the main text, the normalized coupling coefficients for FW and SH are set as $\kappa\left( \mathrm{FW} \right)\approx\kappa\left( \mathrm{SH} \right)=1$.


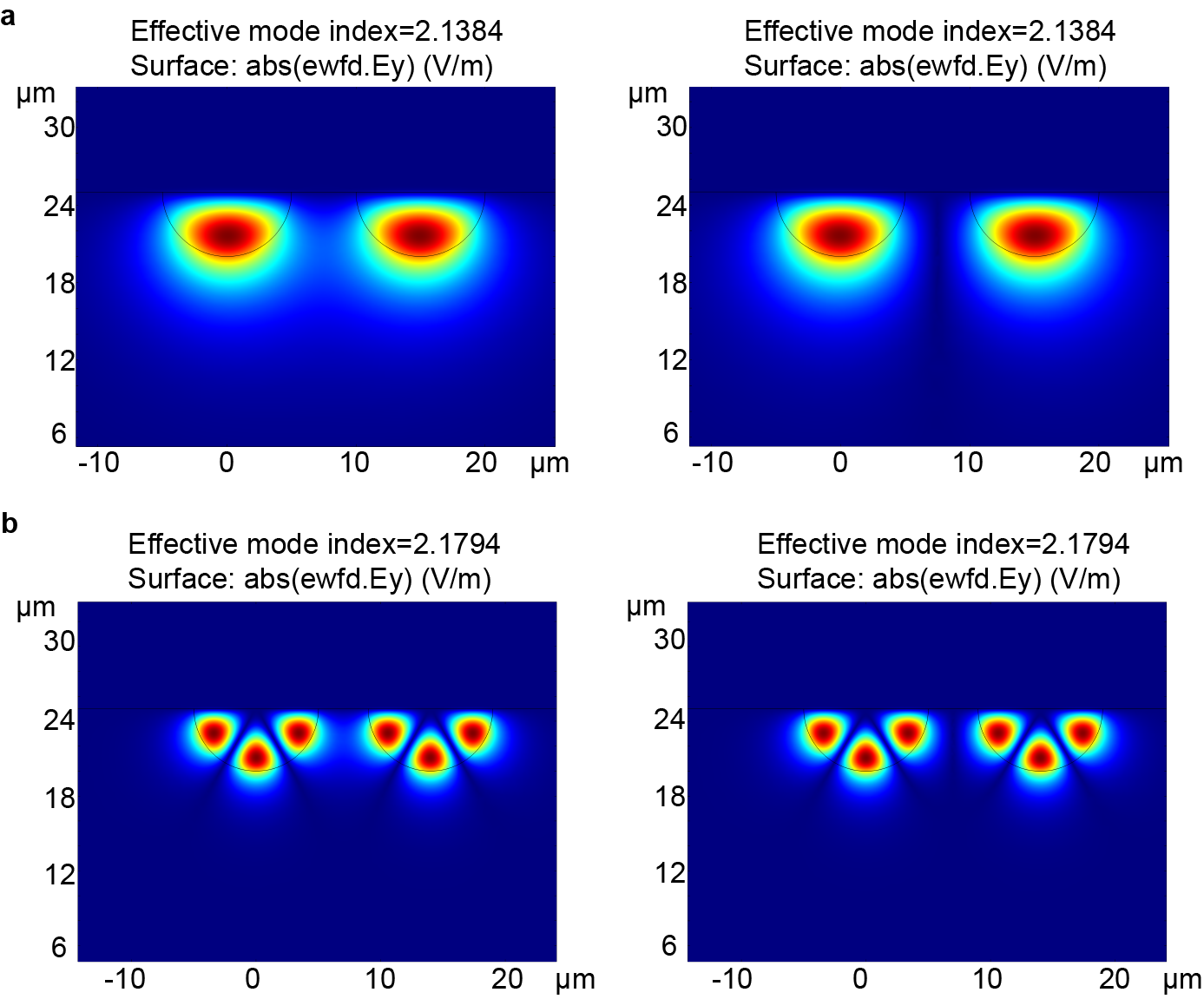


Fig. S2 **a**, Calculated coupled even and odd modes for fundamental wave. **b**, Calculated coupled even and odd modes for second-harmonic wave.

Fig. S1 **a**, Calculated FW fundamental TM_00_ mode profile. **b**, Calculated SH four TM modes profile. **b.1**, TM_00_ mode. **b.2**, TM_01_ mode. **b.3**, TM_02_ mode. **b.4**, TM_10_ mode.


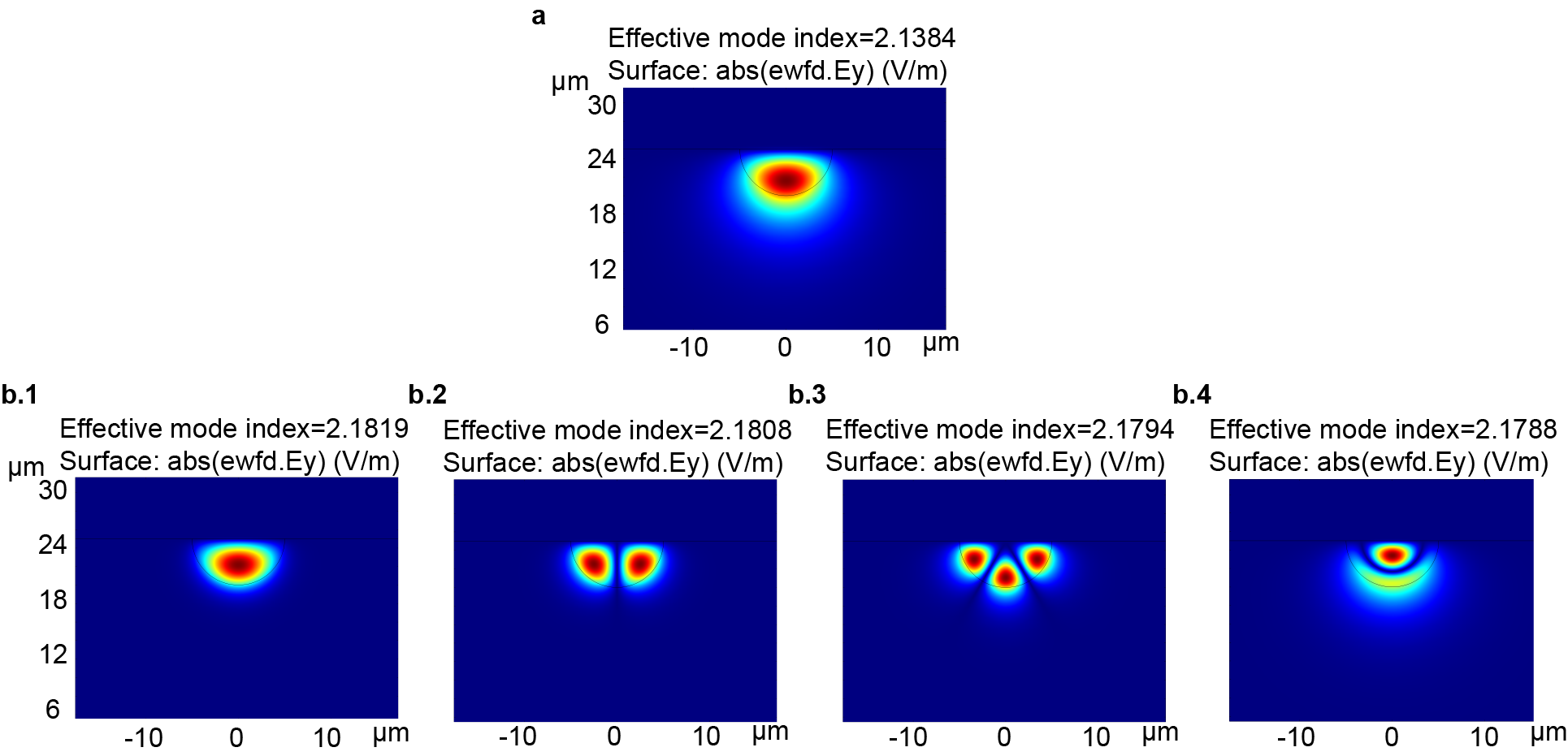


**G. Waveguide design for sum-frequency generation**

In this section, we present a potential design for an optical waveguide array that supports type-0 sum-frequency generation, i.e., $\omega_{1}+\omega_{2}=\omega_{3}$, as described in the main text. Here, the refractive index change from titanium in-diffusion is assumed to be $\Delta n=4\times{10}^{-3}$ and the space distance between waveguide elements is set to be $14 \mu m$. All other parameters are identical to those discussed in Section F. The wavelengths involved in the conversion process are $\lambda_{1}=1.5 \mu m$, $\lambda_{2}=1.3 \mu m$ and $\lambda_{3}=696 \mathrm{nm}$. As shown in Fig. S3, the waveguide channels support a single TM mode at $\lambda_{1}$ and $\lambda_{2}$, while supporting four TM modes at $\lambda_{3}$. Again, due to modal symmetry, only the first, third and fourth modes at $\lambda_{3}$ can interact with the fundamental mode at $\lambda_{1}$ and $\lambda_{2}$. To achieve efficient coupling, quasi-phase matching via periodic poling is employed to phase-match the third mode of $\lambda_{3}$ with the fundamental modes of $\lambda_{1}$ and $\lambda_{2}$. The poling period is given by $\Lambda=\frac{2\pi}{\Delta k}\approx14.3 \mu m$. The small phase mismatch can again be tuned by varying the sample temperature [7].

Fig. S3 Calculated mode profiles of each waveguide channel for **a**, $1500 nm$; **b**, $1300 nm$ and **c**, $696 nm$.


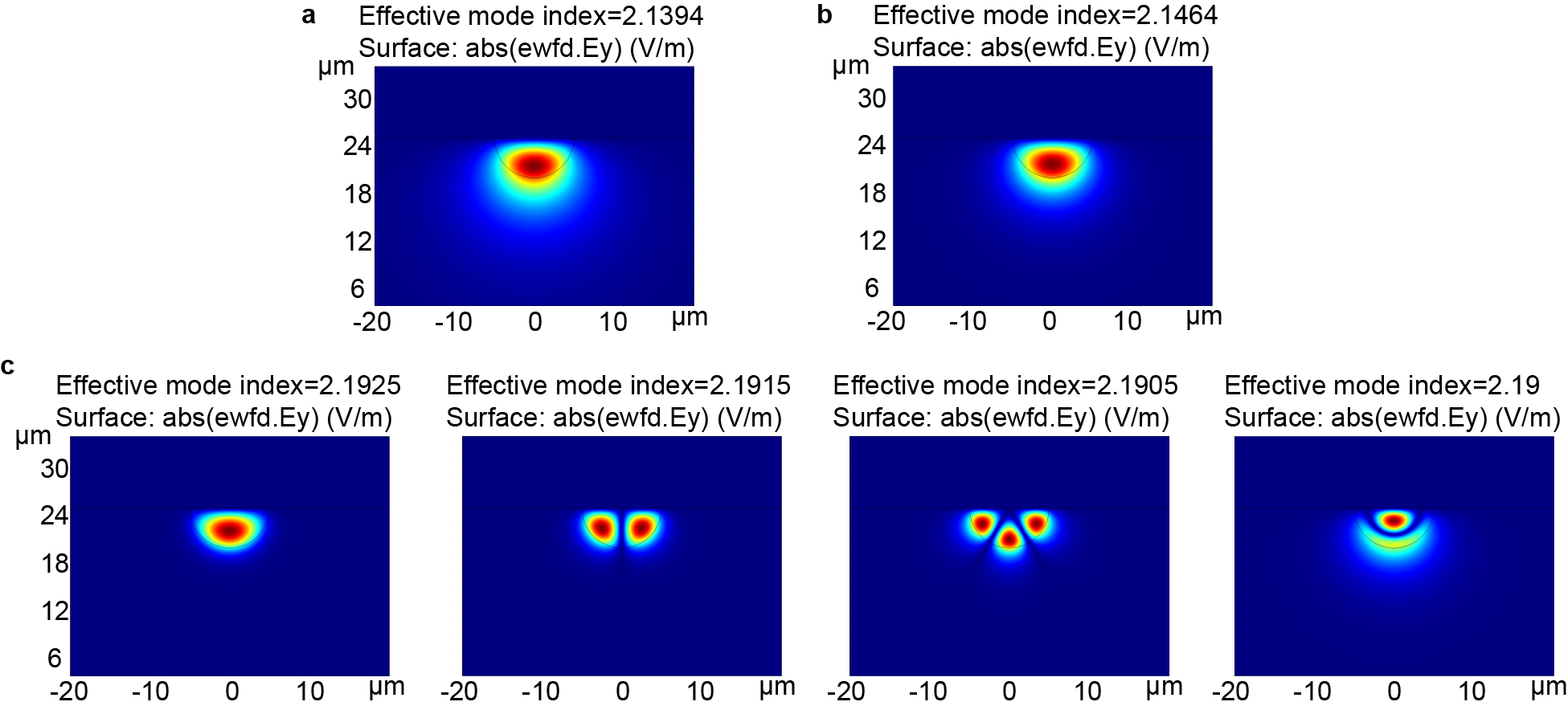


We next calculate the coupling coefficients of these interacting modes. Figure S4 illustrates the coupled even and odd modes at three different frequencies/wavelengths. The corresponding coupling coefficients for each wavelength are presented in Table 1. In the main text, the normalized coupling coefficients are set as $\kappa_{1}=1, \kappa_{2}=0.5,\kappa_{3}=0.2$.

|  | Effective refractive index of even mode | Effective refractive index of odd mode | Coupling coefficient ($\mathbf{m}^{\mathbf{-1}}$) |
| --- | --- | --- | --- |
| $\boldsymbol{\lambda}_{\boldsymbol{1}}$ | 2.139469 | 2.139357 | 234.6 |
| $\boldsymbol{\lambda}_{\boldsymbol{2}}$ | 2.146433 | 2.146387 | 111.2 |
| $\boldsymbol{\lambda}_{\boldsymbol{3}}$ | 2.190465 | 2.190452 | 58.7 |

Table 1. The calculated coupling coefficients for three wavelengths.

Fig. S4 Calculated coupled even and odd modes for wavelengths at **a**, $1500 nm$; **b**, $1300 nm$ and **c**, $696 nm$.


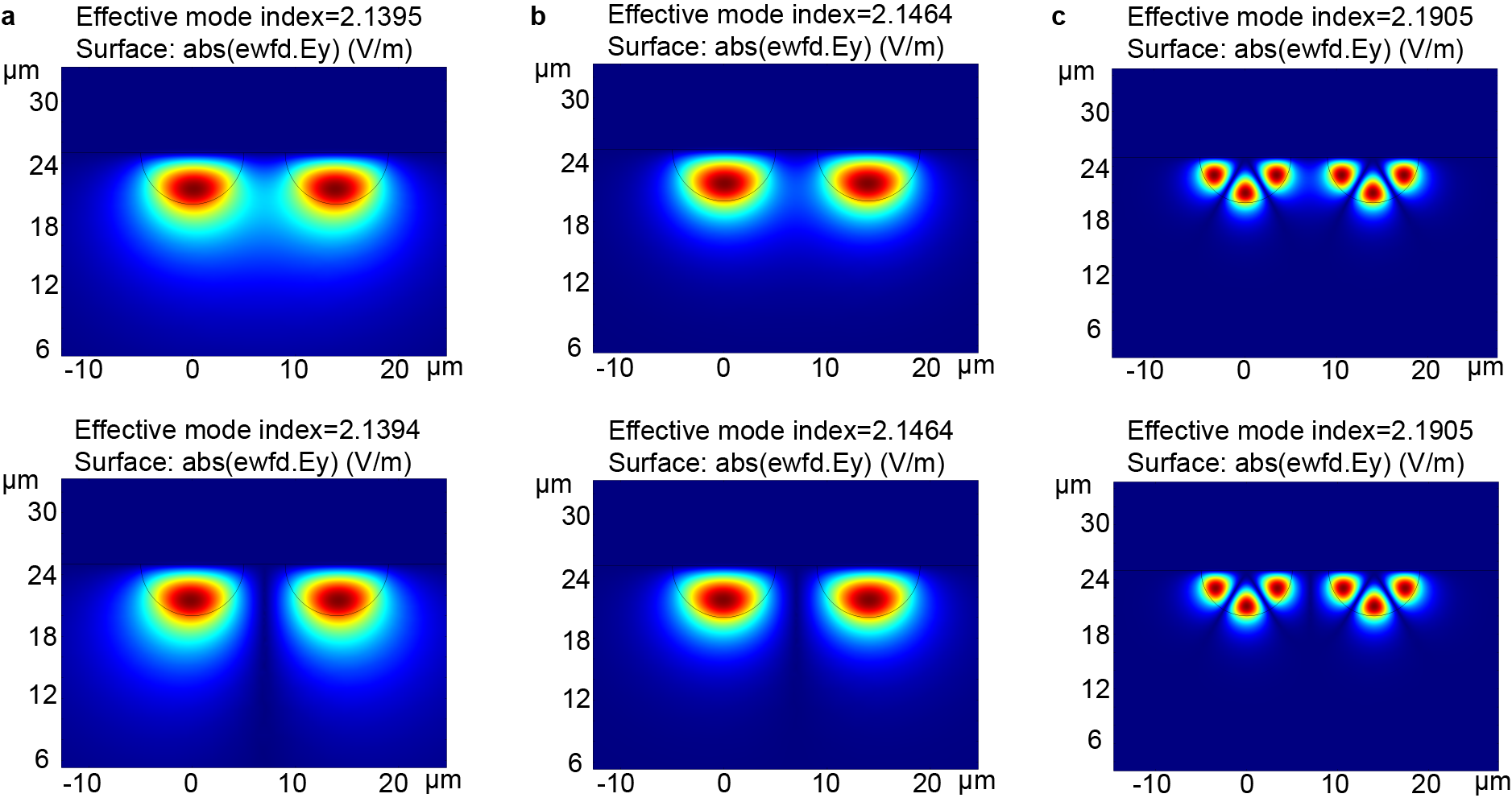


**References:**

1. C. Etrich, F. Lederer, B. A. Malomed, T. Peschel, and U. Peschel, "7 - Optical Solitons in Media with a Quadratic Nonlinearity," in *Progress in Optics*, E. Wolf, ed. (Elsevier, 2000), pp. 483-568.

2. A. Yariv, P. Yeh, and A. Yariv, *Photonics: optical electronics in modern communications* (Oxford university press New York, 2007).

3. K. Okamoto, *Fundamentals of optical waveguides* (Elsevier, 2010).

4. G. A. Siviloglou, K. G. Makris, R. Iwanow, R. Schiek, D. N. Christodoulides, G. I. Stegeman, Y. Min, and W. Sohler, "Observation of discrete quadratic surface solitons," Opt. Express **14**, 5508-5516 (2006).

5. F. Setzpfandt, A. A. Sukhorukov, D. N. Neshev, R. Schiek, Y. S. Kivshar, and T. Pertsch, "Phase Transitions of Nonlinear Waves in Quadratic Waveguide Arrays," Physical Review Letters **105**, 233905 (2010).

6. F. Setzpfandt, A. A. Sukhorukov, and T. Pertsch, "Discrete quadratic solitons with competing second-harmonic components," Phys. Rev. A **84**, 053843 (2011).

7. R. Iwanow, R. Schiek, G. I. Stegeman, T. Pertsch, F. Lederer, Y. Min, and W. Sohler, "Observation of Discrete Quadratic Solitons," Phys. Rev. Lett. **93**, 113902 (2004).
